# Targeting non-coding RNA family members with artificial endonuclease XNAzymes

**DOI:** 10.1038/s42003-022-03987-5

**Published:** 2022-09-24

**Authors:** Maria J. Donde, Adam M. Rochussen, Saksham Kapoor, Alexander I. Taylor

**Affiliations:** Cambridge Institute of Therapeutic Immunology & Infectious Disease (CITIID), Jeffrey Cheah Biomedical Centre, University of Cambridge, Cambridge, UK

**Keywords:** Catalytic DNA, Nucleic acids, Synthetic biology, Antagomir and RNA sponge, miRNAs

## Abstract

Non-coding RNAs (ncRNAs) offer a wealth of therapeutic targets for a range of diseases. However, secondary structures and high similarity within sequence families make specific knockdown challenging. Here, we engineer a series of artificial oligonucleotide enzymes (XNAzymes) composed of 2’-deoxy-2’-fluoro-β-D-arabino nucleic acid (FANA) that specifically or preferentially cleave individual ncRNA family members under quasi-physiological conditions, including members of the classic microRNA cluster miR-17~92 (oncomiR-1) and the Y RNA hY5. We demonstrate self-assembly of three anti-miR XNAzymes into a biostable catalytic XNA nanostructure, which targets the cancer-associated microRNAs miR-17, miR-20a and miR-21. Our results provide a starting point for the development of XNAzymes as a platform technology for precision knockdown of specific non-coding RNAs, with the potential to reduce off-target effects compared with other nucleic acid technologies.

## Abstract (web summary)

Engineered RNA endonuclease XNAzymes can specifically target individual disease-associated non-coding RNAs (ncRNAs) in vitro with potential as platform technology for precision detection or knockdown of individual microRNAs or longer ncRNAs.

## Introduction

The cell is an ‘RNA machine’; although only 1–2% of the human genome encodes peptides, >70% is transcribed, generating a huge variety of non-coding RNAs (ncRNAs) with myriad roles in biology including regulation of chromatin remodelling, mRNA transcription, editing, splicing, translation, and degradation, and are implicated in a wide range of diseases^1,2^. ncRNAs may thus represent a more diverse array of diagnostic biomarkers and therapeutic targets than proteins^3^. microRNAs (miRNAs or miRs) are short (21–25 nts) single-stranded ncRNAs that limit the translation of specific messenger RNAs (mRNAs) by recruitment of the RNA-induced silencing complex (RISC) to miRNA binding sites typically found in the mRNA 3′ untranslated region (UTR), but also in intergenic and intronic regions. miRNA can also interact with other ncRNAs^4^, enabling the formation of highly elaborate post-transcriptional regulatory networks^5–8^. Although initially linked to regulation of development, it has emerged that miRNAs are also key players in hepatitis and cardiovascular disease and can act as both tumour suppressors and oncogenes (oncomiRs) in a variety of cancers^9–11^. From a clinical perspective, the ability of single miRNAs to pleiotropically regulate multiple disease-associated mRNAs – and thus multiple nodes within a given biological pathway (or indeed multiple pathways) – makes them attractive targets for the development of next-generation therapeutics beyond current strategies that intervene at the protein level, against single targets^12–15^.

Current approaches to therapeutic modulation of miRNAs involve small molecule inhibitors or modulators of miRNA processing^16–19^, and a variety of functional oligonucleotide technologies: miRNA activity can be enhanced by delivery of miRNA mimics^20^ into target cells, and can be inhibited by sequestration using transgene-expressed RNAs containing multiple miRNA binding sites (miRNA sponges)^21,22^, or by antisense oligonucleotides (ASOs) that compete for their target sites in mRNAs (blockmiRs), or directly bind to mature miRNAs (antimiR/antagomiRs)^23–25^. However, in addition to the challenges inherent to any oligonucleotide therapeutic technology (delivery, potential immunogenicity, cytotoxicity; reviewed extensively elsewhere^26–28^), several drawbacks to current miRNA modulation approaches remain^29–32^. In principle, such ‘gapmer’ ASOs – oligonucleotides partially modified with synthetic nucleic acid analogues^33^ – recruit host cell RNase H, leading to cleavage and enhanced degradation of RNA targets^27,28^. However, this mechanism is inefficient for mature miRNA targets; antimiR ASOs are thought to principally act by steric blocking and/or sequestration of miRNAs, or inhibition of miRNA processing, rather than induction of RNase H-mediated knockdown^23,34–37^. Indeed, antimiRs have been shown not to reduce the levels of mature miRNAs^38,39^. AntimiRs can thus have limited capacity for multiple-turnover, requiring stoichiometric doses for efficacy. Furthermore, as sufficient basepairing with miRNA targets is necessary for inhibition, antimiR function is typically enhanced by ribose modifications that enable invasion of RNA secondary structure, but which can increase off-target binding and cytotoxicity.

Typical miRNA-inhibiting approaches involve targeting ‘seed regions’, sequence motifs crucial to mRNA binding shared by miRNA family members^40,41^. However, whilst targeting multiple related miRNAs or miRNA binding sites may be appropriate in some circumstances, the increased risk of off-target effects by such strategies remains a key challenge for miRNA therapeutics^15^. Furthermore, the incidence of genetic diseases involving loss of miRNA clusters (such as miR‐17~92 / oncomiR-1^42^, classically implicated in tumorigenesis^43^, but whose deletion is linked to growth defects and arrested B-cell development^44,45^) demonstrates that blanket inactivation of multiple miRNAs can have a range of deleterious consequences. Alternatively, molecular tools that target individual miRNAs would offer a more precise approach to target single ‘nodes’ in densely interconnected regulatory networks.

To this end, targeting miRNAs using oligonucleotide catalysts – ribozymes and DNAzymes^46–49^ – and oligo-peptide conjugates^50–52^, has been suggested. In principle, such ‘antimiRzyme’/‘antagomiRzyme’ catalysts have the potential to be more specific than ASOs due to the combination of substrate-binding arm complementarity and nucleobase requirements at or close to the substrate cleavage site. Furthermore, their capacity to perform enzymatic multiple turnover RNA cleavage independent of host silencing machinery could enable lower dosing and extension of miRNA targeting to settings in which these are unavailable or challenging to recruit, such as extracellular vesicles or exosomes. However, DNAzyme catalysts capable of cleaving all-RNA substrates have proven to be highly dependent on divalent cations for folding, so at physiological concentrations of free Mg^2+^ (<1 mM) – unless co-transfected with divalent metal co-factors^53,54^ – DNAzymes achieve RNA knockdown by triggering ASO-type effects independent of catalytic activity^55–58^, limiting their advantages over other approaches. Extended variants of the hammerhead ribozyme with improved activity under low [Mg^2+^]^59–61^ also offer the prospect of anti-miR catalysts^48^, however, the poor biostability of RNA must be addressed if these are to be developed as exogenously deliverable molecules rather than expressed from virally-delivered transgenes.

**Fig. 1:**
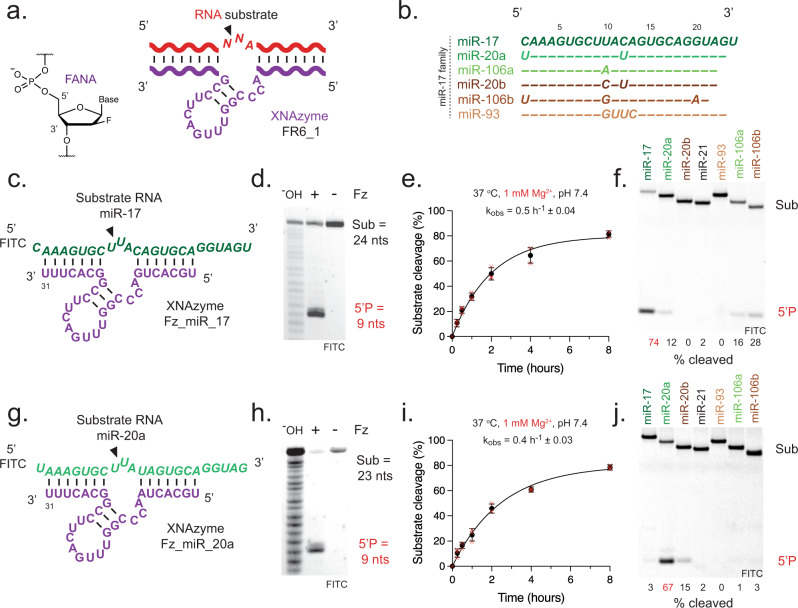
XNAzymes specifically cleave individual members of the miR-17~92 / OncomiR-1 microRNA cluster. **a** Chemical structure of a 2’-deoxy-2’-fluoro-β-D-arabino nucleic acid (FANA) nucleotide and schematic showing the putative secondary structure of the FR6_1 RNA endonuclease XNAzyme composed of FANA (purple) bound to an RNA substrate (red). **b** Sequence similarity between mature microRNAs of the human miR-17 family; note that although alternative sequences have been reported, in this study we use those from ref. ^42^ as proof of concept. Variants of the FR6_1 XNAzyme engineered to cleave (**c**–**f**) miR-17 (“Fz_miR_17”) or (**g**–**j**) miR-20a (“Fz_miR_20a”): (**c**, **g**) putative secondary structures, (**d**–**f**, **h**–**j**) urea-PAGE gels and graphs showing (**d**, **h**) RNA substrate cleavage, (**e**, **i**) catalytic rate constant (k_obs_) and (**f**, **j**) XNAzyme specificity of pseudo first-order single-turnover reactions between cognate miRNA substrates and XNAzymes for (**d**, **h**) 15 h, (**f**, **j**) 5 h, or the times indicated, under quasi-physiological conditions (37 °C, 1 mM Mg^2+^, pH 7.4) (**d**, **e**, **h**, **i**: 1 μM substrate, 5 μM enzyme. **f**, **j** 0.25 μM substrate, 1.25 μM enzyme). Black arrows indicate site of substrate cleavage. (^-^OH) indicates RNA substrates subjected to partial alkaline hydrolysis. Black circles and error bars represent mean and standard error (SEM) for *n* = 3 independent experiments, red crosses represent individual data points.

Analogous “XNAzyme” catalysts can be evolved from a variety of synthetic genetic polymers^62^, including 2′-deoxy-2′-fluoro-β-D-arabino nucleic acid (FANA)^63^, an artificial polymer chemistry capable of stabilising nucleic acid structures and reducing their dependence on Mg^2+^ for folding, and offering improved biostability compared with RNA^64^. We recently identified an artificial modular RNA endonuclease, “FR6_1”, comprising a fully-FANA 17 nt catalytic core flanked by substrate-binding arms (Fig. 1a). Unlike analogous DNAzymes, the FR6_1 XNAzyme retains activity at sub-millimolar Mg^2+^ ^65^, enabling invasion of long, structured RNAs and catalysing site-specific transesterification under physiological conditions^65^.

Here, we explore the potential of XNAzymes to provide highly specific molecular tools for knockdown of disease-associated non-coding RNA. As proof-of-concept, we describe re-targeting the FR6_1 catalyst’s site-specific RNA cleavage activity, establishing a series of all-FANA antimiRzymes / antagomiRzymes tailored to individual human oncomiRs – members of the miR‐17~92 / oncomiR-1 cluster and miR-21 – as well as a ncRNA linked to autoimmune disease, the human Y RNA hY5. We demonstrate that in contrast with previous oligonucleotide catalysts, the resulting XNAzymes have the capacity to efficiently cleave non-coding RNAs under quasi-physiological conditions.

## Results

### Engineering XNAzymes for selective cleavage of oncomiR microRNAs

Firstly, we sought to explore the capacity of XNAzyme FR6_1 to be re-targeted to a variety of classic cancer-associated ‘oncomiR’ microRNAs. Although we have previously found the substrate-binding arms of FR6_1 can be readily redesigned to generate active variants, we have observed sequence preferences for the site of RNA cleavage; although catalysis can be observed with substrates bearing all possible nucleobase combinations immediately adjacent to the cleaved phosphodiester, the XNAzyme is only able to cleave when an adenine is positioned one nucleotide 3′ of this position (A_+1_)(Fig. 1a). We therefore sought to engineer FR6_1 variants to target N^NA motifs (with ^ representing the cleavage site) in mature microRNAs corresponding to members of the miR-17 family in the classic miR-17/92 (oncomiR-1) cluster and its paralogues^42^ (Fig. 1b). One instance of this motif at positions 10-12 (U^UA) in miR-17 is also the principal location of sequence variation between family members (Fig. 1b), which suggested that targeting this region may yield XNAzymes capable of discriminating between them.

Targeting FR6_1 to this position in miR-17 yielded an active XNAzyme, “Fz_miR_17” (Fig. 1c), which site-specifically cleaves a substrate equivalent to the mature miR-17-5p microRNA under quasi-physiological conditions (1 mM Mg^2+^, pH 7.4, 37 °C)(Fig. 1d) with a pseudo-first order pre-steady state catalytic rate constant (k_obs_ = 0.5 h^−1^)(Fig. 1e) comparable to previous re-targeted variants of FR6_1 ^65^. In order to test the specificity of Fz_miR_17, we compared reactions with miR-17, the other members of the miR-17 family (Fig. 1f), and the unrelated miR-21 for 5 h (the end of the steady-state phase of the cognate reaction): although some ‘off-target’ cleavage of miR-106a and miR-106b could be observed, particularly when reactions were allowed to continue to the plateau phase (Supplementary Fig. 1a), Fz_miR_17 showed a clear preference for the cognate target, miR-17 (Fig. 1f). These results demonstrate that substrate specificity may be conferred by: (1) the presence or absence (as in miR-93 (Fig. 1b)) of the required A_+1_, (2) choice of cleavage site dinucleotide (miR-17:U^U is preferred over miR-106a:U^A or miR-106b:U^G (Fig. 1b), consistent with our previous observations^65^) and/or (3) binding arm complementarity, in particular at positions directly flanking the FR6_1 catalytic core – in miR-20a and miR-20b, 12 C > U mutations (Fig. 1b) are predicted to replace the basepair rC12:fG7 (Fig. 1c) with a wobble pair, which reduces activity. Guided by the latter observation, we next prepared a variant of Fz_miR_17 bearing an 7fG > fA mutation in order to make a basepair at this position with miR-20a/b. This yielded “Fz_miR_20a” (Fig. 1g), which was found to be capable of site-specific cleavage of the mature miR-20a under quasi-physiological conditions (1 mM Mg^2+^, pH 7.4, 37 °C)(Fig. 1h) with a similar catalytic rate constant (k_obs_ = 0.4 h^−1^)(Fig. 1i) as Fz_miR_17, and is likewise selective for the cognate microRNA (Fig. 1j), with discrimination between miR-20a and miR-20b due to cleavage site dinucleotide preference (miR-20a:U^U > miR-20b:U^C). Both Fz_miR_17 and Fz_miR_20a were capable of multiple turnover catalysis under quasi-physiological conditions (Supplementary Fig. 1c, d). Encouragingly, both XNAzymes were also able to cleave their cognate microRNAs in both tissue culture media and HEK293 cell lysate (Supplementary Fig. 1e, f).

We also attempted to design a variant of Fz_miR_17 to specifically target miR-106a (Supplementary Fig. 2a) by exploiting this family member’s 11U > A mutation (Fig. 1b), which creates a C^UA motif as a potential cleavage site specific to miR-106a. However, this molecule was found to be inactive under quasi-physiological conditions (Supplementary Fig. 2a). This may be due to stabilisation of a non-catalytic conformation caused by pairing of rC8 in miR-106a with fG24 in the XNAzyme catalytic core (Supplementary Fig. 2a), the equivalent residue to fG27 in the parent FR6_1, which we have previously shown to be crucial for activity. However, it is unclear why another variant of the FR6_1 XNAzyme, “FR6_1NucS^R^”, containing the same core sequence, was previously found to be active on RNA substrate variants bearing an analogous rC, including one with a C^UA motif^65^.

**Fig. 2:**
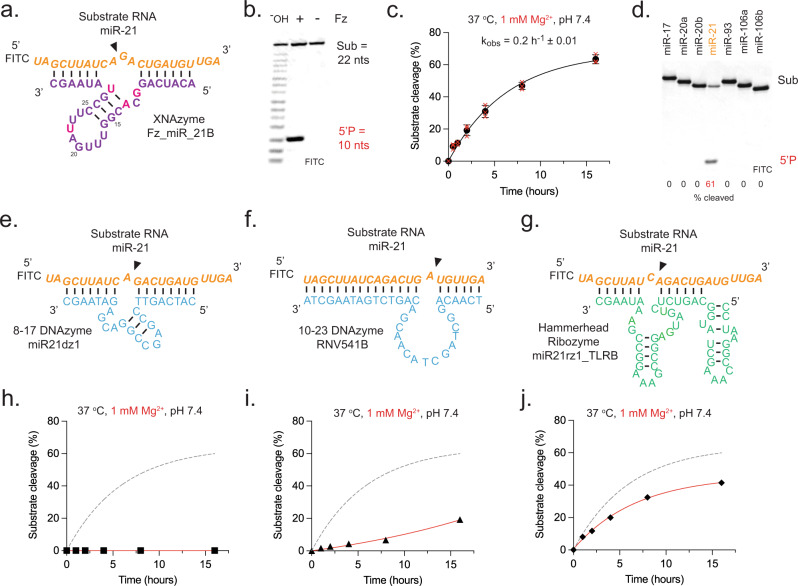
An XNAzyme specifically cleaves microRNA miR-21 and outperforms analogous DNAzyme and ribozyme catalysts. **a** Schematic showing the putative secondary structure of a variant of the FR6_1 XNAzyme, “Fz_miR_21B”, engineered to cleave human miR-21. **b**–**d** Urea-PAGE gels and graphs showing (**b**) substrate cleavage, (**c**) catalytic rate constant (k_obs_) and (**d**) XNAzyme specificity of pseudo first-order single-turnover reactions between (0.5 μM) miR-21 RNA substrate and (2.5 μM) XNAzyme Fz_miR_21B for (**b**, **d**) 15 h, or (**c**) a time series, under quasi-physiological conditions (37 °C, 1 mM Mg^2+^, pH 7.4). (^-^OH) indicates RNA substrates subjected to partial alkaline hydrolysis. Black circles and error bars represent mean and standard error (SEM) for *n* = 3 independent experiments, red crosses represent individual data points. **e**–**g** Schematics showing the putative secondary structure analogous DNAzymes (**e**) “miR21dz1”^48^ and (**f**) “RNV541B”^49^, and (**g**) ribozyme “miR21rz1_TLRB”^48^ (see Supplementary Figs. 3 and 4). **h**–**j** Graphs showing pseudo first-order single-turnover reactions between (0.5 μM) miR-21 RNA substrate and (2.5 μM) (**h**) miR21dz1 (black squares), (**i**) RNV541B (black triangles) or (**j**) miR21rz1_TLRB (black diamonds), under quasi-physiological conditions (37 °C, 1 mM Mg^2+^, pH 7.4). Nucleic acid chemistry is indicated by colour: FANA = purple or (to highlight mutated positions) magenta, RNA = orange (substrate) or green (catalyst), DNA = cyan. Black arrows indicate site of substrate cleavage.

Next, we sought to examine whether specific oncomiR cleavage could be extended beyond the miR-17~92 cluster. miR-21 is an abundant, well-conserved microRNA implicated in the inflammatory response and inhibition of tumour suppressor gene expression. It is thought to play a key role in cardiovascular disease and is the most consistently upregulated miRNA in a range of aggressive cancers including glioblastoma, in which levels of miR-21 significantly correlate with poor prognosis^66^. We designed a variant of FR6_1 re-targeted to a central N^NA motif in the mature miR-21 (residues 10-12; A^GA). Although this variant (“Fz_miR_21”) was found to be only modestly active (~10% cleavage observed after 20 h under quasi-physiological conditions)(Supplementary Fig. 2b), a screen of mutations in the catalytic core (Supplementary Fig. 2c) previously identified in a re-selection experiment to improve activity of a re-targeted FR6_1 variant^65^ revealed a variant, “Fz_miR_21B” (Fig. 2a), bearing four core mutations that led to a ~14-fold improvement in activity (8fA > fG, 10fC > fU, 19fC > fU and 25fG > fU) (Supplementary Fig. 2c). Like the other anti-miR XNAzymes, Fz_miR_21B site-specifically cleaves its target under quasi-physiological conditions (Fig. 2b), with a comparable catalytic rate constant (k_obs_ = 0.2 h^−1^)(Fig. 2c), and is highly selective for miR-21 (Fig. 2d) and capable of multi-turnover catalysis (Supplementary Fig. 2d).

In order to benchmark the anti-miR XNAzymes to other microRNA-targeting oligonucleotide catalysts designed to cleave at, or close to, the same site targeted by Fz_miR_21B, we also sought to examine the cleavage of miR-21 using previously described DNAzymes based on 8-17 or 10-23 catalysts (Supplementary Fig. 3), “miR21dz1”^48^ (Fig. 2e) and “RNV541”^49^, respectively, or an extended variant of the hammerhead ribozyme (Supplementary Fig. 4), “miR21rz1_TLR”^48^, containing a ‘tetraloop receptor’ (TLR) motif previously shown to improve activity in low [Mg^2+^]^67^. However, although DNAzyme miR21dz1 was found to be active under high magnesium conditions (10 mM Mg^2+^), both DNAzyme RNV541 and ribozyme miR21rz1_TLR were found to be inactive (Supplementary Figs. 3 and 4). Having identified possible errors in the published sequences, we synthesised variants, “RNV541B” (Fig. 2f) and “miR21rz1_TLRB” (Fig. 2g), with mutations that restored activity (Supplementary Figs. 3 and 4).

Although miR-21 cleavage could be observed in high magnesium (10 mM [Mg^2+^]), catalytic activity of DNAzymes miR21dz1 and RNV541B was substantially reduced or entirely abolished at quasi-physiological magnesium concentrations (1 mM Mg^2+^)(Supplementary Fig. 3). Ribozyme miR21rz1_TLRB activity was likewise perturbed upon reduction of [Mg^2+^] (Supplementary Fig. 4), as indeed was Fz_miR_21B activity (Supplementary Fig. 3). Nevertheless, in 1 mM [Mg^2+^] the Fz_miR_21B XNAzyme out-performed both analogous 8-17 and 10-23 DNAzymes (Fig. 2c, h, i) and the analogous hammerhead ribozyme (Fig. 2j).

We also compared the stability of Fz_miR_21B (FANA), miR21dz1 (DNA), RNV541B (DNA) (Supplementary Fig. 3) and miR21rz1_TLRB (RNA) (Supplementary Fig. 4). Consistent with our previous findings^65^, Fz_miR_21B (which was not further modified with, for example, phosphorothioate linkages in the backbone^68^) exhibited a comparable rate of degradation (half-life ~2 h) as RNV541B (~1.5 h) in 50% human serum at 37 °C, whereas miR21dz1 and miR21rz1_TLRB were degraded within minutes or seconds (Supplementary Figs. 3 and 4).

### Engineering XNAzymes for selective cleavage of hY5 non-coding RNA

Encouraged by the capacity of the FR6_1 XNAzyme to generate catalysts tailored to specific microRNAs, we wondered whether this could be extended to longer, more structured non-coding RNAs (ncRNAs). Y RNAs are a family of highly conserved molecules involved in assembly of RNA-protein complexes with a variety of roles including DNA replication and RNA stability and quality control^69^. Recently, Y RNAs were found to be among a set of ncRNAs that can be glycosylated and expressed on the cell surface^70^, potentially providing a range of ligands and receptors with as yet unknown roles in biology and disease – and may shed light on the involvement of non-coding RNA as antigens in automimmune disease; autoantibodies against the Y RNA hY5, for example, can be detected in systemic lupus erythematosus (SLE)^71^.

**Fig. 3:**
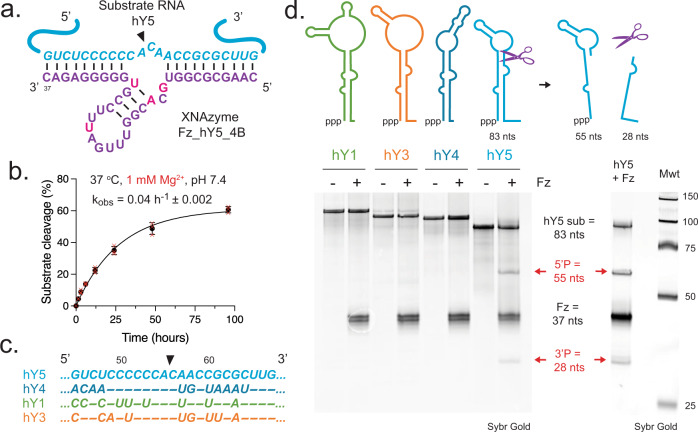
An XNAzyme specifically cleaves non-coding Y RNA hY5. **a** Schematic showing the putative secondary structure of a variant of the FR6_1 XNAzyme, “Fz_hY5_4B”, engineered to cleave human non-coding Y RNA hY5. FANA residues are indicated in purple or (to highlight mutated positions) magenta. Substrate hY5 RNA is shown in cyan. Black arrow indicates site of substrate cleavage. **b** Graph showing catalytic rate constant (k_obs_) of a pseudo first-order single-turnover reaction between (0.5 μM) hY5 RNA substrate and (2.5 μM) XNAzyme Fz_hY5_4B under quasi-physiological conditions (37 °C, 1 mM Mg^2+^, pH 7.4). Black circles and error bars represent mean and standard error (SEM) for *n* = 3 independent experiments, red crosses represent individual data points. **c** Sequence similarity of human Y RNAs at the target site of XNAzyme Fz_hY5_4B. **d** Schematics and urea-PAGE gels showing specificity of XNAzyme Fz_hY5_4B in pseudo first-order single-turnover reactions between (0.5 μM) Y RNA substrates and (2.5 μM) XNAzyme Fz_hY5_4B under quasi-physiological conditions (24 h, 37 °C, 1 mM Mg^2+^, pH 7.4). Note that unglycosylated versions of the Y RNAs were used in this study as the site and chemical nature of glycosylation linkage to these RNAs is currently unknown^70^.

In order to explore the possibility of precise targeting such molecules, we chose hY5 as proof of concept and engineered and screened a series of FR6_1 variants targeting a subset of N^NA motifs, informed by preferences for cut site dinucleotide combinations (Supplementary Fig. 5a). Two designs (“Fz_hY5_4” and “Fz_hY5_5”) were found to be weakly active (Supplementary Fig. 5b). Transferring the same set of catalytic core mutations found to improve activity of Fz_miR_21B (Fig. 2a) into one of these, Fz_hY5_4, yielded “Fz_hY5_4B” (Fig. 3a), an XNAzyme targeted to hY5 residues 46-68 and cleaving between residues 56 and 57 (Fig. 2a)(Supplementary Fig. 5), albeit with a more modest (~10-fold slower) catalytic rate constant (k_obs_ = 0.04 h^−1^) (Fig. 3b) than the anti-miR XNAzymes. Despite the extensive similarity between hY5 and the other Y RNA family members, the Fz_hY5_4B target site contains an A_+1_ residue that is unique to hY5 (U in hY1/3/4) (Fig. 3c), enabling Fz_hY54B-induced cleavage to be limited specifically to this family member and target sequence. Incubation of Fz_hY5_4B with hY5 RNA (in quasi-physiological conditions) therefore results in cleavage into two RNA products of the expected electrophoretic mobility (55 nts and 28 nts), but no reaction with the other human Y RNAs (Fig. 3d).

### Assembly of a catalytic FANA nanostructure targeting multiple microRNAs

Next, we sought to explore whether multiple ncRNA-targeting XNAzymes could be combined into a single multi-functional artificial enzyme. We have previously found that XNAs including FANA can be engineered to self-assemble into simple nanoscale polyhedra^72^. In principle, nanostructure designs can improve biostability by sterically hindering access to 3′ termini by serum exonucleases and promote cellular uptake of nucleic acid catalysts^73^, and could enable synergistic targeting of multiple microRNAs^74^.

**Fig. 4:**
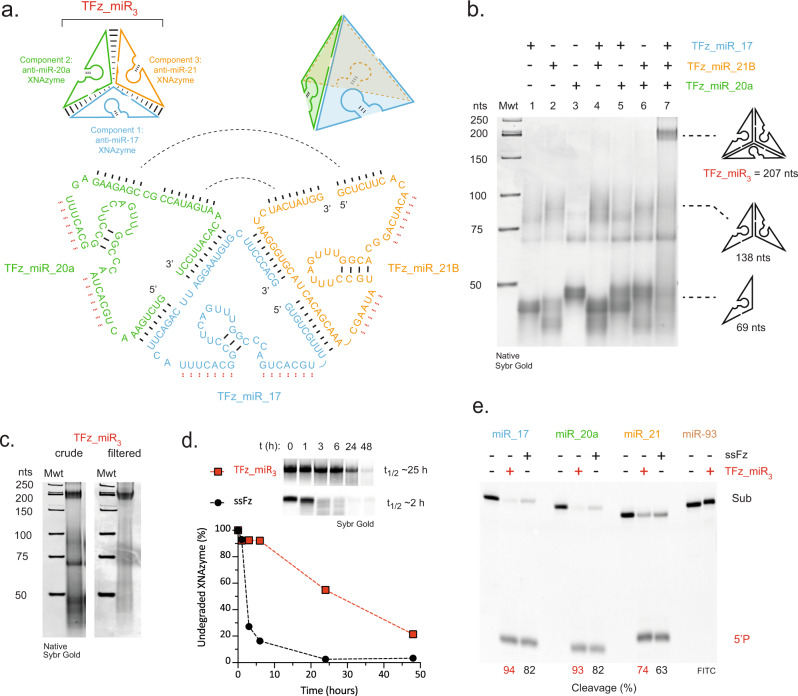
A catalytic XNA nanostructure has improved biostability and targets multiple microRNAs. **a** Schematics showing design of a three-component fully-FANA tetrahedron (“TFz_miR_3_”) presenting three different anti-miR XNAzymes (Fz_miR_17, Fz_miR_20a and Fz_miR_21B) along single-stranded edges (strand hybridisation is designed to be mediated by base pairing indicated by black lines; red lines indicate residues mediating microRNA substrate binding). **b** Native PAGE gel showing self-assembly of the compete TFz_miR_3_ tetrahedron (lanes 1-3: individual TFz_miR components, lanes 4-6: combinations of two TFz_miR components, lane 7: all three TFz_miR components). Mwt = NEB low molecular weight ladder (dsDNA). **c** Native PAGE gels showing crude or spin filter purified TFz_miR_3_ (following 1 h at 37 °C). **d** Urea-PAGE gels and graph showing stability of the assembled and purified TFz_miR_3_ (red squares) or a single-stranded XNAzyme (“ssFz”)(Fz_miR_21B)(black circles) in 50% human serum at 37 °C. **e** Urea-PAGE gels showing activity of (2.5 μM) assembled and purified TFz_miR_3_ or the appropriate single-stranded XNAzyme (Fz_miR_17, Fz_miR_20a or Fz_miR_21B) in pseudo first-order single-turnover reactions with (0.5 μM) cognate RNA substrates or an untargeted control miRNA (miR-93) under quasi-physiological conditions (24 h, 37 °C, 1 mM Mg^2+^, pH 7.4).

In order to examine whether this could be applied to the anti-miR XNAzymes, we sought to modify the three component sequences of a DNA tetrahedron design previously described by Hollenstein, Ahn et al.^73^ that presents three variants of the 17E DNAzyme on single-stranded edges of the nanostructure. The 17E DNAzyme sequences in the component strands (‘S3’, ‘S4’ and ‘S5’ in ref. ^73^) were replaced with those of the three anti-miR XNAzymes and the complete sequence of each component strand synthesised as fully-FANA oligonucleotides (“TFz_miR_17”, “TFz_miR_20a” and “TFz_miR_21B”) – in principle each component strand contains sequences that hybridise to the other strands, enabling self-assembly into the complete 3D catalytic nanostructure (“TFz_miR_3_”) (Fig. 4a). Examination of self-assembly by non-denaturing native PAGE (Fig. 4b) revealed that, in isolation, components were somewhat heterogenous, yielding smeared bands with electrophoretic mobility equivalent to a mixture of monomers and homodimers, which exhibit identical mobility on denaturing page (Supplementary Fig. 6). However, an electrophoretic mobility shift equivalent to the assembled tetrahedron (207 nts) was observed when all three components were annealed (Fig. 4b).

Following purification of fully-assembled TFz_miR_3_ by removal of the majority (~75%) of any remaining monomers or dimers by centrifugal filtration (Fig. 4c), we examined the biostability and catalytic activity of the nanostructure. TFz_miR_3_ exhibited a ~20-fold enhanced half-life relative to single-stranded FANA XNAzymes in human serum (Fig. 4d) and was found to retain the activity of all three anti-miR XNAzymes, cleaving mir-17, miR-20a and miR-21 under quasi-physiological conditions (Fig. 4e).

## Discussion

Despite the growing variety of strategies for modulation of disease-associated microRNAs, most approaches rely on sequestering or sterically blocking their activity - i.e. non-enzymatic mechanisms with limited capacity for multiple turnover - or by recruiting host RNA silencing mechanisms, which can be inefficient for knockdown of microRNAs compared with mRNA targets, and may be limiting in some cell types or compartments. Although antimiR oligonucleotide designs that bind shared seed sequences in order to target entire miRNA families may be beneficial in some circumstances, specificity for single microRNAs remains challenging; longer complementary sequences or chemical modifications that enhance T_m_ of RNA binding in order to invade miRNA secondary structure increase the likelihood of binding to other RNAs in the transcriptome, leading to off-target knockdown or inhibition, as well as saturation and competition effects on endogenous RNA processing machinery^29^. Platforms that enable individual ncRNAs to be targeted among highly similar sequences, and do not rely on co-opting host factors for activity, could offer new opportunities for precise modulation of RNA networks.

Starting from a previously-described RNA endonuclease XNAzyme, FR6_1^65^, we demonstrate that a variety of oligonucleotide enzymes can be engineered to cleave a range of ncRNAs, enabling highly specific targeting of individual members of both short (miRNAs of the miR-17~92 cluster) and longer (Y RNA) ncRNA families. In contrast with analogous DNA and RNA catalysts, whose activity in vitro is typically only measurable under unrealistic conditions (high concentrations of Mg^2+^ (10–25 mM) and catalyst (100 μM))^46,48,49^, we show that the anti-ncRNA XNAzymes efficiently cleave their targets under quasi-physiological conditions (1 mM Mg^2+^) and at modest concentrations (0.5 μM)(Supplementary Figs. 1 and 2). Although miRNA knockdown (or upregulation of miRNA targets) in vivo has been reported following transfection with anti-miR ribozymes and DNAzymes^46,48,49,75^, it is unclear whether these observations derive from antisense-mediated, steric hinderance or cytotoxic effects (as is the case for analogous catalysts targeting mRNAs^55–58^). Observations that anti-miR DNAzyme activity is reduced or abolished under physiologically-relevant conditions in vitro^46,48,49^, as we confirm here for two reported anti-miR-21 catalysts, is consistent with an in vivo knockdown mechanism that does not depend upon catalytic turnover. Our finding that the anti-ncRNA XNAzymes we describe significantly outperform such catalysts (at least in vitro) provides a promising starting point for investigation of their activity in vivo, although we note that dissecting the relative contribution of bona fide catalysis and antisense-mediated effects to intracellular activity can be challenging^65,76^.

Although the catalytic rates of the ncRNA-targeting XNAzymes were found to be relatively modest (k_obs_ = 0.04 - 0.5 h^−1^), with generally reduced activity compared with the parental FR6_1 catalyst (k_obs_ = 1.5 h^−1^)^65^ under quasi-physiological conditions, these could in principle be further improved by ‘maturation’ selections or mutation screens (as we demonstrate here in the case of the anti-miR-21 XNAzyme). The rates of microRNA cleavage catalysed by XNAzymes nonetheless compare favourably with the rate of mature microRNA turnover in vivo, which can be very slow (half-lives of 10–30 h)^77^, suggesting that specific cleavage at k_obs_ ≥ 0.04 h^−1^ ought to be, at least in principle, sufficient to modulate intracellular miRNA levels.

Although we did not explore the capacity of XNAzymes to cleave miRNA transcripts and/or precursor miRNAs, which are processed to yield mature miRNAs^5,8^, targeting the latter may be in any case a more valuable strategy for knockdown of individual miRNAs, particularly in light of observations that mature miRNA levels do not rapidly decline following targeting of precursor miRNAs by anti-miRs^48^, inhibition of transcription^78^ or deletion of processing machinery^79^. A key outstanding question is whether XNAzymes have the capacity to cleave mature miRNAs in the RISC complex, as the majority of mature microRNAs are associated with Argonaute proteins. In the case of anti-miR ASOs, the mechanism of miRNA inhibition notably involves binding to complexed miRNAs but not their ejection from Argonaute nor a reduction of their levels^38,39^. However, small RNAs with highly complementarity to target miRNAs induce unloading from Argonaute^80^, suggesting that, with careful optimisation of XNAzyme composition and design, XNAzyme-induced ejection ought to be possible if indeed dissociation from RISC is required for miRNA cleavage.

Whilst intracellular conditions indeed support FR6_1 XNAzyme-mediated catalysis, as we examine elsewhere^65^ and consistent with our observations here with anti-miR XNAzymes in cell lysate (Supplementary Fig. 1e, f), in any case anti-miR XNAzymes would require delivery to the cytoplasm, which we have yet to address. However, it bodes well for the future development of this technology that ASOs composed of FANA have been shown to be capable of self-delivery without transfection reagents^81–83^. Furthermore, the capacity of XNAzymes to assemble into catalytic nanostructures, as we explore here, offers the possibility to exploit this strategy for delivery^73,84^.

The FANA chemistry comprising the FR6_1-derived XNAzymes – a DNA analogue whose fluorinated arabinose sugar enables highly stable homo- and heteroduplex formation^64,68,85,86^ – likely provides the ncRNA-targeting XNAzymes with an enhanced capacity to invade RNA target secondary structure (although this likely trades off with increased product inhibition). Furthermore, FANA, as we have explored using the parental FR6_1 catalyst, likely stabilises intramolecular pairings in the catalytic core, reducing dependency on divalent cations for folding of the catalytically-active tertiary structure. However, high affinity FANA:FANA pairings in the core may also enhance the proportion of misfolded catalytically-inactive XNAzymes, as suggested by the plateauing of pseudo first-order reactions at 60 - 80% cleavage.

Future elaboration of catalysts could involve modified nucleobases with the potential to evolve multiple catalytic mechanisms^87^ and/or sugar- or backbone-modified chemistries with advantageous biostability and pharmacodynamics^88^. The use of nucleic acid analogues capable of stabilising secondary structures is an established approach to improve RNA target accessibility in the context of microRNA-targeting agents; 2′-O-methyl-RNA (2′OMe-RNA), 2′-methoxyethyl-RNA (2′MOE-RNA) and locked nucleic acids (LNA) have proven to be of particular utility for antimiR and antagomiR development^23,89–91^ and likewise enhance the activity of nucleic acid catalysts^46,47,92,93^. Recently, synthetic genetic systems capable of efficient synthesis and reverse transcription of these chemistries have been established^94,95^, and the elaboration of RNA-cleaving catalysts demonstrated using fully-2OMe-RNA polymers^95^. These systems thus enable the chemical structure space of 2′OMe-RNA, 2′MOE-RNA and LNA to be explored for the development of antimiRzyme / antagomiRzyme XNAzymes with enhanced biostability, RNA structure invasion and cleavage activity in physiological conditions. Furthermore, short LNAs are capable of superior target strand mismatch discrimination^96,97^, which has been exploited to reduce siRNA off-target effects^98^, offering a route to XNAzymes with further improvements in substrate specificity. However, the capacity for multiple-turnover catalysis can be limited by extensive modification with such chemistries^99^, so careful consideration must be given to optimise this in the context of ncRNA target binding.

Our results extend the range of targets of artificial RNA endonuclease XNAzymes to include disease-associated non-coding RNA and provide a proof-of-concept demonstration, at least in vitro, of their potential utility as highly specific tools for detection or knockdown of individual microRNAs or longer ncRNAs.

## Methods

DNA and RNA (and chimeric DNA/RNA) oligonucleotides were synthesized by Integrated DNA technologies (Belgium) or Sigma-Aldrich, MilliporeSigma (USA). Polymerase D4K^100^ was kindly provided by Philipp Holliger (MRC Laboratory of Molecular Biology, Cambridge). HEK293 cells were kindly provided by Jake Barker (Dept. of Pathology, University of Cambridge).

### Preparation of XNAzymes and RNA substrates

FANA XNAzymes were synthesized enzymatically, using DNA/RNA primer “drP2_Ebo”, 3′ biotinylated DNA templates (e.g. “Fz_miR_17_temp”; Supplementary Table 1), triphosphates of FANA (faNTPs)(Metkinen Chemistry, Finland), and polymerase D4K, as described previously^62,101^; single-stranded XNAzymes were subsequently purified by capturing (biotinylated) DNA templates using streptavidin magnetic beads (Dynabeads MyOne C1, Thermo Fisher Scientific, USA) and eluting (unbiotinylated) XNAzymes using 0.1 N sodium hydroxide, followed by removal of the drP2_Ebo DNA/RNA primer by hydrolysis by incubation in 0.7 N NaOH for 1 hr at 65 °C.

Y RNA substrates were prepared by annealing the relevant oligonucleotides shown in Supplementary Table 1 (e.g. for synthesis of hY5 RNA: hY1tempFwd and hY1tempRev) to form double stranded templates for RNA synthesis using an SP6 transcription kit (NEB, USA), according to the manufacturer’s instructions.

XNAzymes and RNA substrates were purified by denaturing urea-PAGE and ethanol-precipitated from filtrates of freeze-thawed gel mash. Prior to initiation of reactions XNAzymes and RNA substrates were annealed in nuclease-free water (Qiagen, Germany) by incubation at 80 °C for 60 s, then cooled to room temperature for 5 min.

Partial alkaline hydrolysis of RNA substrates for use as electrophoresis size standards was performed by incubation at 65 °C in 20 mM sodium hydroxide (pH 12) for 4 min, then neutralized with 1 M Tris pH 7.

### Preparation of cell lysate

HEK293T cells were grown in Dulbecco’s Modified Eagle Medium (DMEM) (MilliporeSigma, USA) supplemented with 10% foetal calf serum (FCS) (Gibco, Thermo Fisher Scientific, USA) and 100 U/ml penicillin G, 0.1 mg/mL streptomycin and 2 mM L-glutamine (Gibco, Thermo Fisher Scientific, USA) in 5% CO_2_ at 37 °C. Cells were detached with StemPro Accutase (Gibco, Thermo Fisher Scientific, USA), resuspended in fresh DMEM, washed twice with PBS, once with ‘Mg-free cell wash buffer’ (25 mM EPPS pH 7.4, 140 mM KCl) and the supernatant removed. Cell pellets were lysed by 6 cycles of freeze-thawing in a dry ice ethanol bath and lysate filtered through 0.2 μm Spin-X filtration columns (Thermo Fisher Scientific, USA). Lysates were heated at 95 °C for 10 min to inactivate RNases.

### Determination of XNAzyme activity

PAGE-purified XNAzymes and RNA substrates were annealed separately as described above and reacted in ‘quasi-physiological buffer’ (30 mM EPPS pH 7.4, 150 mM KCl, 1 mM MgCl_2_) at 37 °C. Reactions were stopped by addition of PAGE gel loading buffer (95% formamide, 20 mM Tris pH 7.5, 10 mM EDTA, 0.05% bromophenol blue) and analysed by Urea-PAGE using 20% acrylamide gels. Gels containing fluorophore-labelled RNA substrates and 5’ RNA products were imaged without staining using an FLA-5000 scanner (Fujifilm, Japan); unlabelled RNA substrates and products were first stained using SYBR Gold stain (Thermo Fisher Scientific, USA). Bands were quantified using Fiji^102^ to calculate proportion of RNA cleaved for each XNAzyme reaction. For SYBR Gold stained RNA products, a coefficient to account for differential staining proportional to oligo length was applied to product band densities. Pseudo first-order reaction rates (k_obs_) under single-turnover pre-steady-state (K_m_/k_cat_) conditions were determined from time courses; samples were taken and reactions stopped at appropriate intervals by snap-freezing on dry ice in excess PAGE gel loading buffer. Quantification data from three independent replicates per time course were fit using Prism 9 (GraphPad), as described previously^62^.

### Preparation of XNA nanostructures

The sequences for the FANA tetrahedron were adapted from components “S3”, “S4” and “S5” of a DNA tetrahedron described by Thai et al.^73^. FANA component strands for the catalytic tetrahedron (“TFz_miR_3_”), “TFz_miR_17”, “TFz_miR_20a” and “TFz_miR_21B”, were prepared from appropriate DNA templates (Supplementary Table 1) and primers removed by hydrolysis as described above. PAGE-purified component strands were mixed in an equimolar ratio in nuclease-free water (Qiagen, Germany) and annealed by incubation at 95 °C for 2 min then cooled to 15 °C at 0.1 °C/s. Tetrahedra were purified using 50KDa ultra centrifugal filter concentrator (Amicon, MilliporeSigma, USA) as recommended by the manufacturer with three 10 min wash steps.

### Biostability assays

PAGE-purified TFz_miR_3_ tetrahedron or single-stranded catalysts were annealed as described above, then incubated (at 0.5 uM final) at 37 °C in 50% human serum (MilliporeSigma, USA) for 48 h. Samples were taken and reactions stopped at appropriate intervals by snap-freezing on dry ice in excess PAGE gel loading buffer. The proportion of undegraded full-length catalyst was determined by Urea-PAGE; gels were stained with SYBR Gold (Thermo Fisher Scientific, USA) and imaged as described above.

### Statistics and reproducibility

All XNAzyme reactions were replicated at least three times using different batches of FANA. Statistical analyses of kinetics experiments were performed on three independent replicates, defined as separate reactions using separately annealed catalysts and substrates.

### Reporting summary

Further information on experimental design is available in the Nature Research Reporting Summary linked to this paper.

## Supplementary information

Supplementary Information

Description of Additional Supplementary Data

Supplementary Data 1

Reporting Summary-New

## Data Availability

All data generated or analysed during this study are included in this published article (and its supplementary information files; raw gel images including those used to generate graphs are provided in “Supplementary Data 1”) or are available upon reasonable request.
