# Supplementary material for: Targeting non-coding RNA family members with artificial endonuclease XNAzymes
Source: Commun Biol. 2022 Sep 24;5:1010. doi: 10.1038/s42003-022-03987-5 (PMC9509326; doi:10.1038/s42003-022-03987-5)
Supplement: Supplementary file 1 — Supplementary Information [file 42003_2022_3987_MOESM1_ESM.pdf]

Supplementary Information for:

Targeting non-coding RNA family members with artificial endonuclease XNAzymes

Maria J. Donde[1], Adam M. Rochussen[1], Saksham Kapoor[1] and Alexander I. Taylor[1]*

[1]Cambridge Institute of Therapeutic Immunology & Infectious Disease (CITIID), Jeffrey Cheah Biomedical Centre, University of Cambridge, Cambridge, UK.

*corresponding author: ait29@cam.ac.uk

Contents:

Supplementary Figures 1-6.
Supplementary Table 1.
Supplementary References.

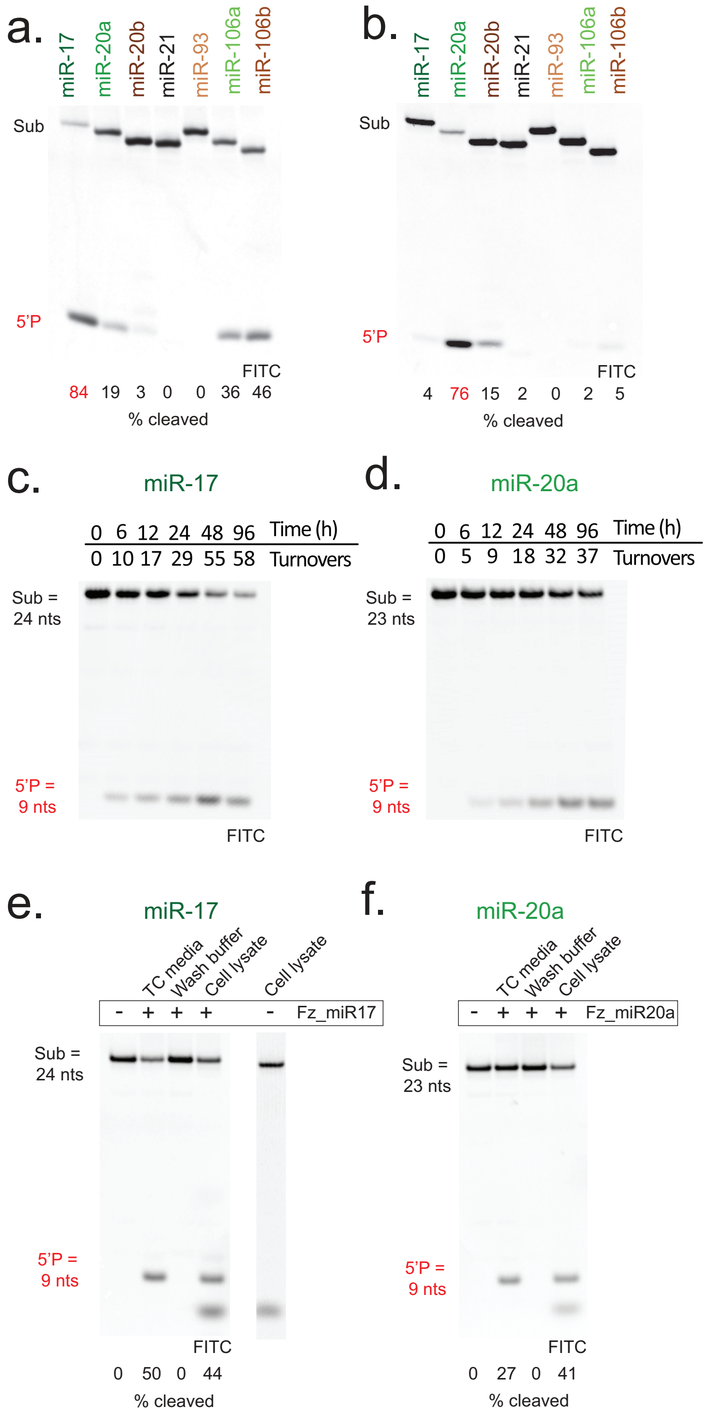

Supplementary Figure 1. Further characterisation of XNAzymes targeted to microRNAs.

*Urea-PAGE gels showing (**a,b**) single-turnover reactions between (0.25 μM) microRNA substrates and 1.25 μM XNAzyme (a) Fz_miR_17 or (b) Fz_miR_20a, for 8h under quasi-physiological conditions (37 °C, 1 mM Mg^{2+}, pH 7.4). (**c,d**) multiple-turnover reactions between (50 μM)(c) miR-17 or (d) miR-20a RNA substrates and (0.5 μM) XNAzyme (c) Fz_miR_17 or (d) Fz_miR_20a, under quasi-physiological conditions (37 °C, 1 mM Mg^{2+}, pH 7.4). (**e,f**) single-turnover reactions between (0.5 μM) (e) miR-17 or (f) miR-20a and (2.5 μM) (e) Fz_miR_17 or (f) Fz_miR_20a, in DMEM tissue culture media (4 h), Mg-free cell wash buffer (5 h) or 90% HEK293 cell lysate (5 h).*

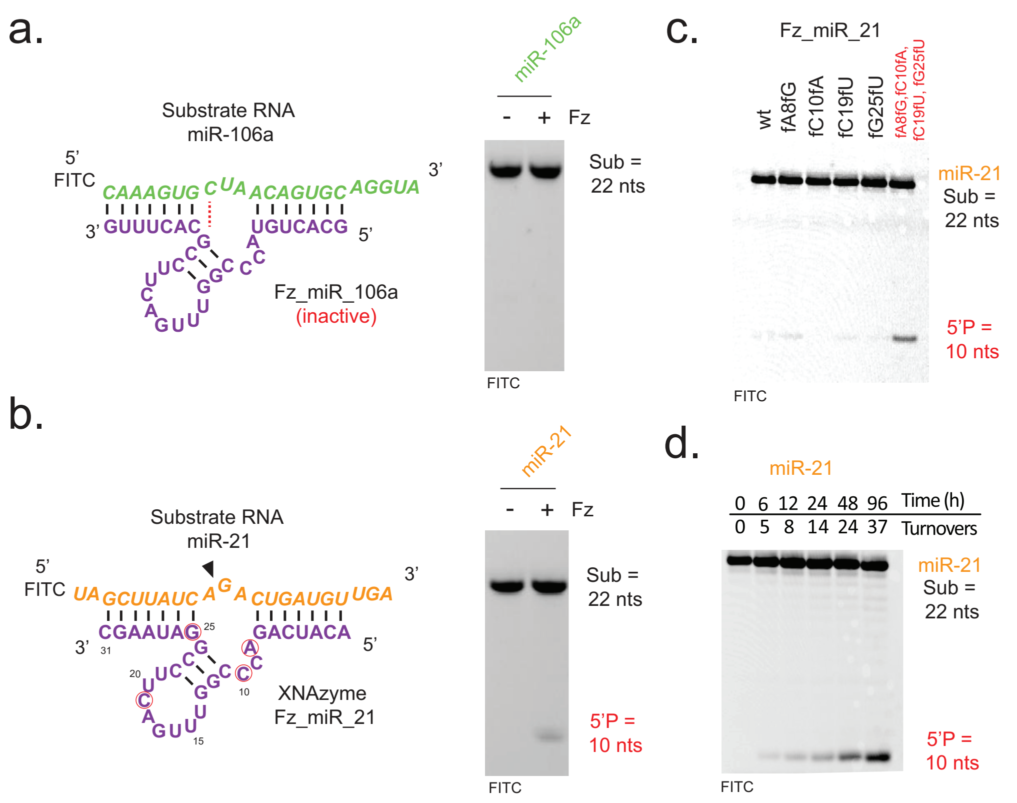

Supplementary Figure 2. Engineering XNAzymes targeted to miR-106a and miR-21.

*(**a**) Schematic and urea-PAGE gel showing putative secondary structure and (lack of) activity of a variant of the FR6_1 XNAzyme, "Fz_miR_106a", engineered to cleave human miR-106a. No detectable product was observed after 15 h with (1 μM) miR-106a RNA substrate and (5 μM) XNAzyme Fz_miR_106a. Dashed red line indicates possible complementarity responsible for destabilising secondary structure in the catalytic core. (**b**) Schematic and urea-PAGE gel showing putative secondary structure and activity of a variant of the FR6_1 XNAzyme, "Fz_miR_21", engineered to cleave human miR-21. Black arrows indicate site of substrate cleavage. (1 μM) miR-21 RNA substrate and (5 μM) XNAzyme Fz_miR_21 were incubated for 15 h. Red circles indicate positions mutated in variants of Fz_miR_21 and (**c**) screened for improved activity; (1 μM) miR-21 RNA substrate and (5 μM) each XNAzyme Fz_miR_21 variant were incubated for 2 h. (**d**) Urea-PAGE gel showing multiple-turnover reaction between (50 μM) miR-21 RNA substrate and (0.5 μM) XNAzyme "Fz_miR_21B" [Fz_miR_21: fA8fG, fC10fA, fC19fU, fG25fU]. All reactions shown were performed under quasi-physiological conditions (37 °C, 1 mM Mg^{2+}, pH 7.4).*

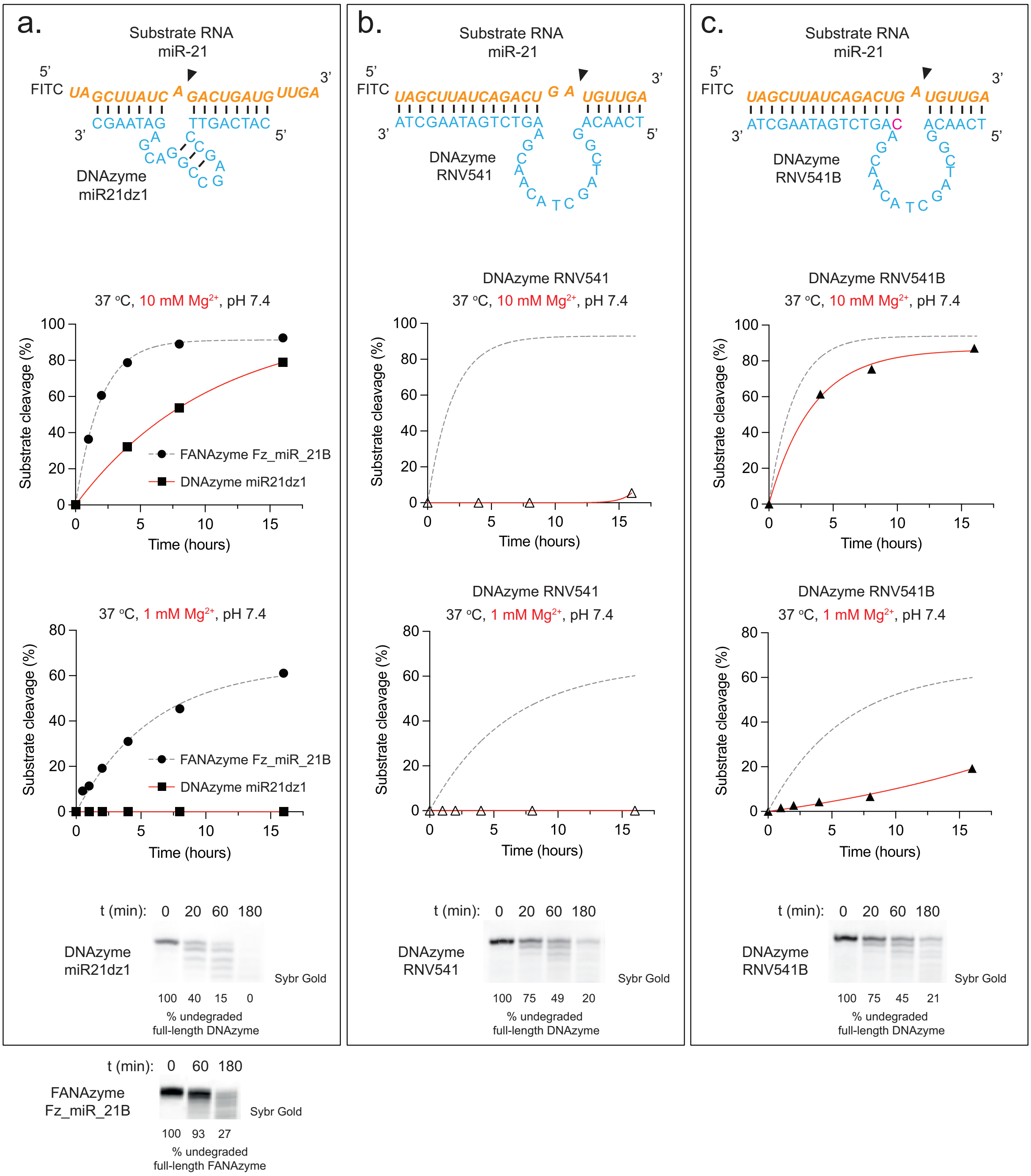

Supplementary Figure 3. Characterisation of DNAzymes targeted to miR-21.

*(**a-c**) Schematics showing DNAzymes targeting miR-21 based on (a) the 8-17 DNAzyme, "miR21dz1"* [1]*, and (b,c) the 10-23 DNAzyme, "RNV541"* [2]*, graphs showing their RNA-cleavage activity in pseudo first-order single-turnover reactions between (0.5 μM) miR-21 RNA substrate and (2.5 μM) DNAzyme, in 'high magnesium' conditions (37 °C, 10 mM Mg^{2+}, pH 7.4), or quasi-physiological conditions (37 °C, 1 mM Mg^{2+}, pH 7.4) (Solid red lines indicate DNAzyme data. Dashed grey lines indicate data for analogous reactions with FANA XNAzyme Fz_miR_21B), and Urea-PAGE gels showing their degradation in 50 % human serum at 37 °C (with data for FANA XNAzyme Fz_miR_21B shown for comparison). Black arrows indicate site of substrate cleavage. DNA residues are indicated in cyan. Substrate RNA residues are indicated in orange.*

DNAzyme "RNV541" (b) is the sequence as published by Larcher et al.[2]*, but as this sequence had little activity in our hands, a variant (c) "RNV541B" was also synthesised with an inserted dC (indicated in red) to restore canonical binding arm pairing with the miR-21 RNA substrate.*

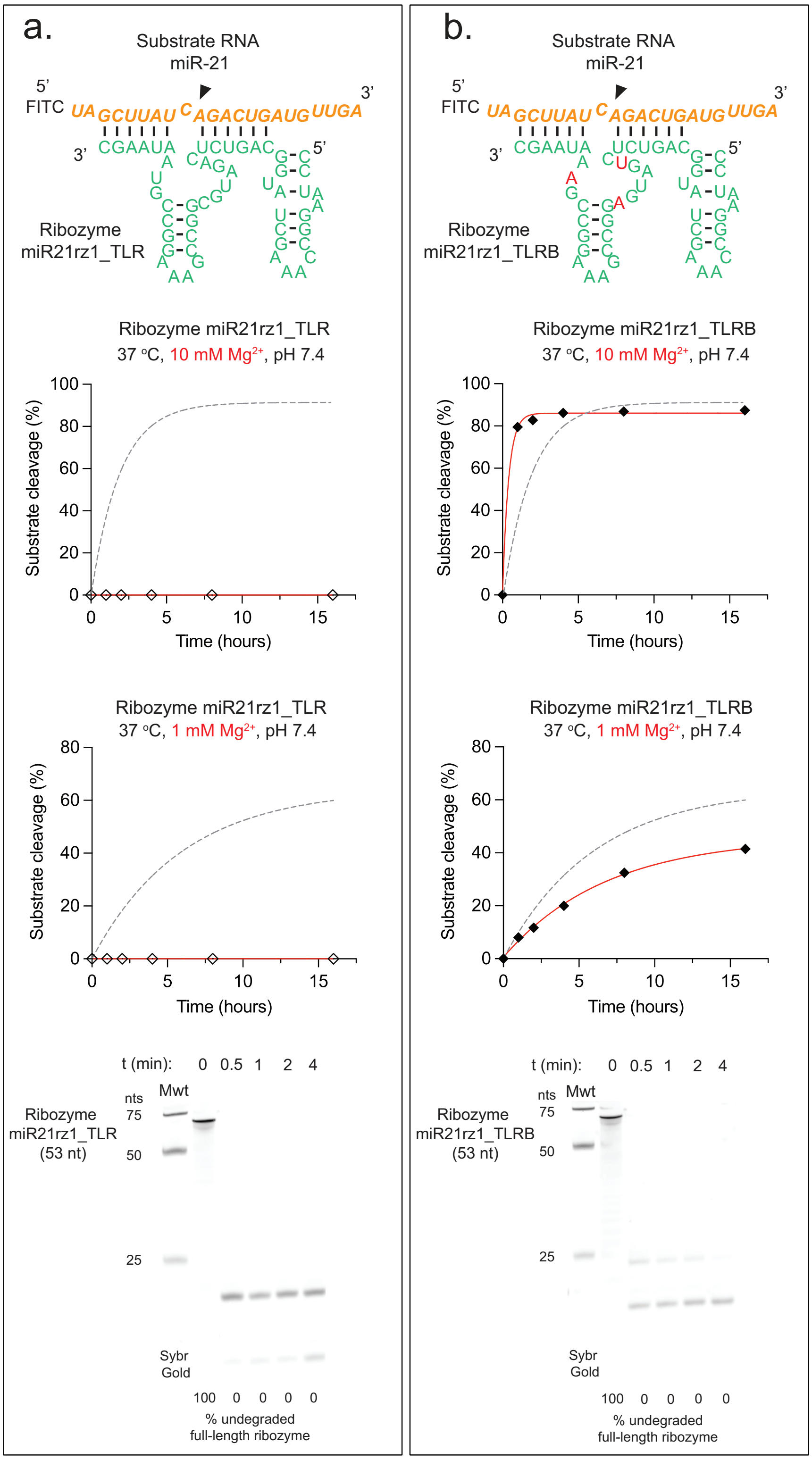

Supplementary Figure 4. Characterisation of ribozymes targeted to miR-21.

*(**a,b**) Schematics showing ribozymes targeting miR-21 based on the extended hammerhead ribozyme, "miR21rz1_TLR" [1], graphs showing their RNA-cleavage activity in pseudo first-order single-turnover reactions between (0.5 μM) miR-21 RNA substrate and (2.5 μM) ribozyme, in 'high magnesium' conditions (37 °C, 10 mM Mg^{2+}, pH 7.4), or quasi-physiological conditions (37 °C, 1 mM Mg^{2+}, pH 7.4) (Solid red lines indicate DNAzyme and ribozyme data. Dashed grey lines indicate data for analogous reactions with FANA XNAzyme Fz_miR21B), and Urea-PAGE gels showing their degradation in 50 % human serum at 37 °C. Black arrows indicate site of substrate cleavage. Catalyst RNA residues are indicated in green. Substrate RNA residues are indicated in orange.*

Ribozyme "miR21rz1_TLR" (a) is the sequence as published by Belter et al.[1] but as this sequence was found to be inactive in our hands, a variant (b) "miR21rz1_TLRB" with three mutations (indicated in red) was synthesised that restores the canonical hammerhead ribozyme core sequence.

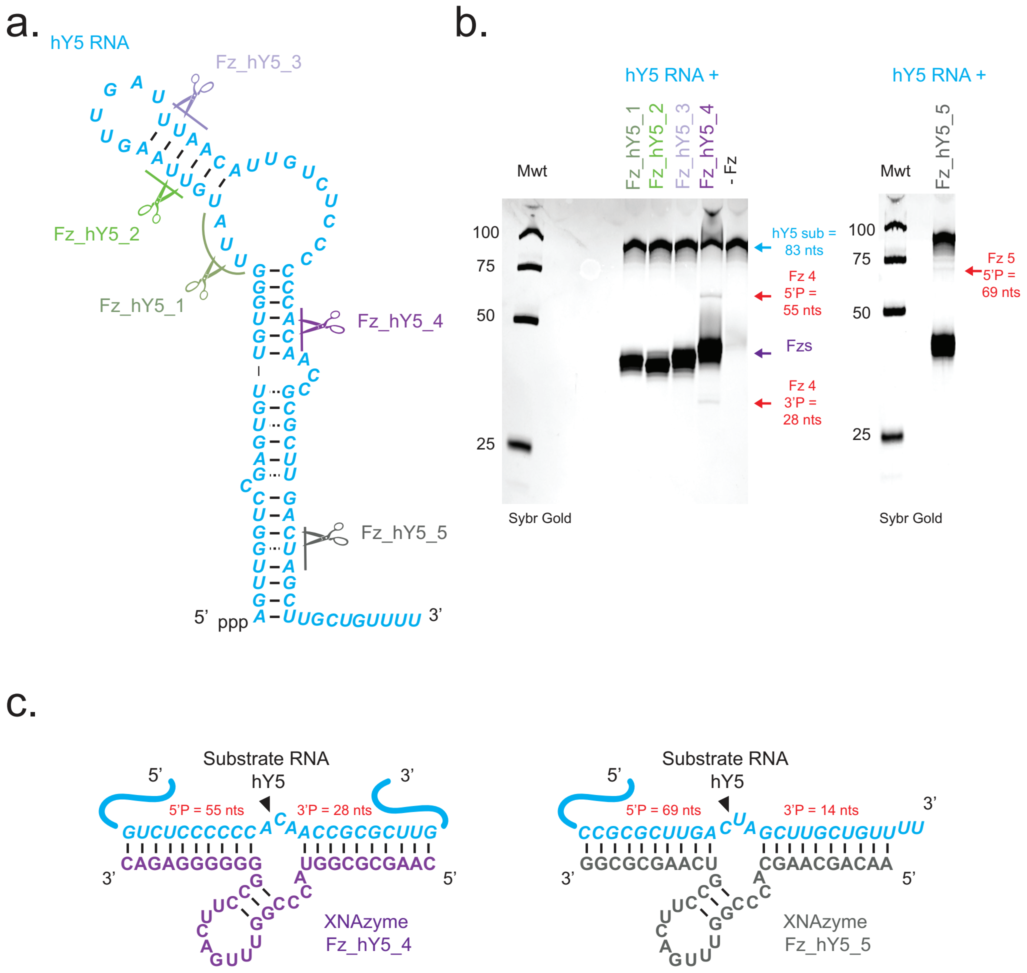

Supplementary Figure 5. Screening XNAzymes targeted to non-coding Y RNA hY5.

*(**a**) Schematic showing the secondary structure of human hY5 RNA indicating potential cleavage site motifs targeted by a series of variants of the FR6_1 XNAzyme, "Fz_hY5_[1-5]". (**b**) Urea-PAGE gels showing activity of XNAzymes Fz_hY5_[1-5] in pseudo first-order single-turnover reactions between (0.5 μM) hY5 RNA substrate and (2.5 μM) XNAzymes under quasi-physiological conditions (37 °C, 1 mM Mg^{2+}, pH 7.4s) for 15 hrs. Bands with PAGE mobility corresponding to the expected molecular weight of cleavage products could be observed in reactions with (**c**) XNAzymes "Fz_hY5_4" and "Fz_hY5_5". Black arrows indicate site of substrate cleavage.*

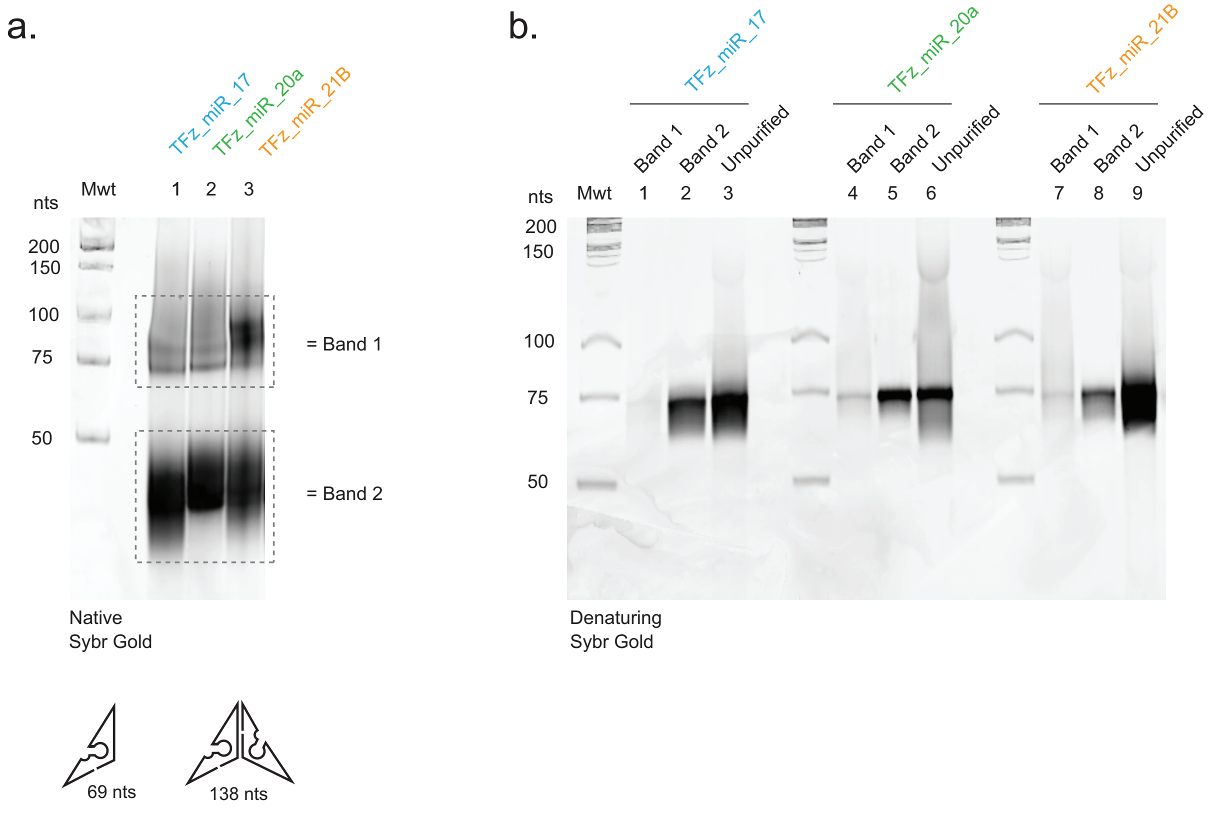

Supplementary Figure 6. Electrophoretic mobility of TFz_miR$_3$ tetrahedron components in native and denaturing PAGE.

*(**a**) Native PAGE gel showing annealed individual FANA component strands of the TFz_miR$_3$ tetrahedron. For each component, bands indicated by dotted line boxes were extracted and re-analysed by (**b**) Denaturing Urea-PAGE. Mwt = NEB low molecular weight ladder (dsDNA).*

Name	Sequence (5' – 3')	Description
drP2_Ebo	[6FAM] AATCTACCACATCGCTCATTG	Primer for synthesis of XNAzymes (3' RNA residue allows primer removal after synthesis)
Fz_miR_17temp	AAAGTGCCGGAAGTCAAACCGGGTCAGTGCACAA TGAGCGATGTGGTAGATT [BiotinTEG]	Template for synthesis of FANAzyme Fz_miR_17
Fz_miR_20atemp	AAAGTGCCGGAAGTCAAACCGGGTTAGTGCACAA TGAGCGATGTGGTAGATT [BiotinTEG]	Template for synthesis of FANAzyme Fz_miR_20a
Fz_miR_106atemp	CAAAGTGCGGAAGTCAAACCGGGTACAGTGCCAA TGAGCGATGTGGTAGATT [BiotinTEG]	Template for synthesis of Fz_miR_106a
Fz_miR_21temp	GCTTATCCGGAAGTCAAACCGGGTCTGATGTCAA TGAGCGATGTGGTAGATT [BiotinTEG]	Template for synthesis of FANAzyme Fz_miR_21
Fz_miR_21[A8G]temp	GCTTATCCGGAAGTCAAACCGGGCCTGATGTCAA TGAGCGATGTGGTAGATT [BiotinTEG]	Template for synthesis of FANAzyme Fz_miR_21 with A8G mutation
Fz_miR_21[C10A]temp	GCTTATCCGGAAGTCAAACCGTGTCTGATGTCAAT GAGCGATGTGGTAGATT [BiotinTEG]	Template for synthesis of FANAzyme Fz_miR_21 with C10A mutation
Fz_miR_21[C19U]temp	GCTTATCCGGAAATCAAACCGGGTCTGATGTCAA TGAGCGATGTGGTAGATT [BiotinTEG]	Template for synthesis of FANAzyme Fz_miR_21 with C19U mutation
Fz_miR_21[G25U]temp	GCTTATACGGAAGTCAAACCGGGTCTGATGTCAA TGAGCGATGTGGTAGATT [BiotinTEG]	Template for synthesis of FANAzyme Fz_miR_21 with G25U mutation
Fz_miR_21Btemp	GCTTATACGGAAATCAAACCGTGCCTGATGTCAAT GAGCGATGTGGTAGATT [BiotinTEG]	Template for synthesis of FANAzyme Fz_miR_21B (Fz_miR_21: A8G, C10A, C19U & G25U)
miR21dz1	CATCAGTTCCGAGCCGGACGAGATAAGC	8-17 DNAzyme targeting miR-21 described by Belter *et al.*[1]
RNV541	TCAACAGGCTAGCTACAACGAAGTCTGATAAGCT A	10-23 DNAzyme targeting miR-21 described by Larcher *et al.*[2]
RNV541B	TCAACAGGCTAGCTACAACGACAGTCTGATAAGC TA	10-23 DNAzyme targeting miR-21 described by Larcher *et al.*[2], with insertion C22 (underlined)
miR21rz1_TLR	CCUAAGGCCAAAGCUAUGGCAGUCUCAGAUGC GGCCGAAAGGCCGUAAUAAGC	Extended hammerhead ribozyme targeting miR-21 described by Belter *et al.*[1]
miR21rz1_TLRB	CCUAAGGCCAAAGCUAUGGCAGUCUCUGAUGA GGCCGAAAGGCCGAAAUAAGC	Extended hammerhead ribozyme targeting miR-21 described by Belter *et al.*[1], with mutations A27U, C32A, U46A (underlined)
Fz_hY5_1temp	GTGTTGTGGGCGGAAGTCAAACCGGGTTTGTTAA GTTCAATGAGCGATGTGGTAGATT [BiotinTEG]	Template for synthesis of Fz_hY5_1
Fz_hY5_2temp	TGGGTTATTGCGGAAGTCAAACCGGGTAGTTGAT TTACAATGAGCGATGTGGTAGATT [BiotinTEG]	Template for synthesis of Fz_hY5_2
Fz_hY5_3temp	TTAAGTTGATCGGAAGTCAAACCGGGTACATTGT CTCCAATGAGCGATGTGGTAGATT [BiotinTEG]	Template for synthesis of Fz_hY5_3
Fz_hY5_4temp	GTCTCCCCCCCGGAAGTCAAACCGGGTACCGCGC TTGCAATGAGCGATGTGGTAGATT [BiotinTEG]	Template for synthesis of FANAzyme Fz_hY5_4
Fz_hY5_5temp	CCGCGCTTGACGGAAGTCAAACCGGGTGCTTGCT GTTCAATGAGCGATGTGGTAGATT [BiotinTEG]	Template for synthesis of FANAzyme Fz_hY5_5
Fz_hY5_4Btemp	GTCTCCCCCACGGAAATCAAACCGTGCACCGCGC TTGCAATGAGCGATGTGGTAGATT [BiotinTEG]	Template for synthesis of FANAzyme Fz_hY5_4B

		(Fz_hY5_4: A11G, C13A, C22U & G28U)
TFz_miR_17temp	CGTGGGAAGCACATTCCTAAGTCTGAAGTAAAGTGCCGGAAGTCAAACCGGGTCAGTGCAAAACGACACCAATGAGCGATGTGGTAGATT [BiotinTEG]	Template for synthesis of FANAzyme Fz_miR_17 with sequences for tetrahedron assembly [analogous to ‘S3’ in ref [3]]
TFz_miR_21Btemp	CCATAGTAGATTCCCACGTAGTGTCGTTTGCTTATACGGAAATCAAACCGTGCCTGATGTGTGAAGAGCCAATGAGCGATGTGGTAGATT [BiotinTEG]	Template for synthesis of FANAzyme Fz_miR_20B with sequences for tetrahedron assembly [analogous to ‘S5’ in ref [3]]
TFz_miR_20atemp	AGGAATGTGTTACTATGGCGGCTCTTCTCAAAGTGCCGGAAGTCAAACCGGGTTAGTGCAGTTTCAGACCAATGAGCGATGTGGTAGATT [BiotinTEG]	Template for synthesis of FANAzyme Fz_miR_20a with sequences for tetrahedron assembly [analogous to ‘S4’ in ref [3]]
miR-17	[6FAM] CAAAGUGCUUACAGUGCAGGUAGU	Mature miR-17 substrate NB: representative sequences of the miR-17~92 cluster (taken from ref [4]).
miR-20a	[6FAM] UAAAGUGCUUAUAGUGCAGGUAG	Mature miR-20a substrate
miR-20b	[6FAM] CAAAGUGCUCAUAGUGCAGGUA	Mature miR-20b substrate
miR-21	[6FAM] UAGCUUAUCAGACUGAUGUUGA	Mature miR-21 substrate
miR-93	[6FAM] CAAAGUGCUGUUCGUGCAGGUAG	Mature miR-93 substrate
miR-106a	[6FAM] CAAAGUGCUAACAGUGCAGGUA	Mature miR-106a substrate
miR-106b	[6FAM] UAAAGUGCUGACAGUGCAGAU	Mature miR-106b substrate
hY1tempFwd	ATTTAGGTGACACTATAGGCTGGTCCGAAGGTAGTGAGTTATCTCAATTGATTGTTCACAGTCAGTTACAGATCGAACTCCTTGTTCTACTCTTTCCCCCCTTCTCACTACTGCACTTGACTAGTCTTTT	Template for synthesis of hY1 RNA substrate using SP6
hY1tempRev	AAAAGACTAGTCAAGTGCAGTAGTGAGAAGGGGGGAAAGAGTAGAACAAGGAGTTCGATCTGTAACTGACTGTGAACAATCAATTGAGATAACTCACTACCTTCGGACCAGCCTATAGTGTCACCTAAAT	Template for synthesis of hY1 RNA substrate using SP6
hY3tempFwd	ATTTAGGTGACACTATAGGCTGGTCCGAGTGCAGTGGTGTTTACAACTAATTGATCACAACCAGTTACAGATTTCTTTGTTCCTTCTCCACTCCCACTGCTTCACTTGACTAGCCTTTT	Template for synthesis of hY3 RNA substrate using SP6
hY3tempRev	AAAAGGCTAGTCAAGTGAAGCAGTGGGAGTGGAGAAGGAACAAAGAAATCTGTAACTGGTTGTGATCAATTAGTTGTAAACACCACTGCACTCGGACCAGCCTATAGTGTCACCTAAAT	Template for synthesis of hY3 RNA substrate using SP6
hY4tempFwd	ATTTAGGTGACACTATAGGCTGGTCCGATGGTAGTGGGTTATCAGAACTTATTAACATTAGTGTCACTAAAGTTGGTATACAACCCC CCACTGCTAAATTTGACTGGCTTTTT	Template for synthesis of hY4 RNA substrate using SP6
hY4tempRev	AAAAAGCCAGTCAAATTTAGCAGTGGGGGGTTGTATACCAACTTTAGTGACACTAATGTTAATAAGTTCTGATAACCCACTACCATCGGACCAGCCTATAGTGTCACCTAAAT	Template for synthesis of hY4 RNA substrate using SP6
hY5tempFwd	ATTTAGGTGACACTATAGTTGGTCCGAGTGTTGTGGGTTATTGTTAAGTTGATTTAACATTGTCTCCCCCCACAACCGCGCTTGACTAGCTTGCTGTTT	Template for synthesis of hY5 RNA substrate using SP6
hY5tempRev	AAACAGCAAGCTAGTCAAGCGCGGTTGTGGGGGGAGACAATGTTAAATCAACTTAACAATAACCCACAACACTCGGACCAACTATAGTGTCACCTAAAT	Template for synthesis of hY5 RNA substrate using SP6

Supplementary Table 1. Oligonucleotide sequences used in the study.

Black and red and text indicates DNA and RNA sequences respectively.

Supplementary References

1 Belter, A. *et al.* Inhibition of miR-21 in glioma cells using catalytic nucleic acids. *Scientific Reports* **6**, 24516, doi:10.1038/srep24516 (2016).

2 Larcher, L. M., Wang, T. & Veedu, R. N. Development of Novel antimiRzymes for Targeted Inhibition of miR-21 Expression in Solid Cancer Cells. *Molecules* **24**, doi:10.3390/molecules24132489 (2019).

3 Thai, H. B. D. *et al.* Tetrahedral DNAzymes for enhanced intracellular gene-silencing activity. *Chemical Communications* **54**, 9410-9413, doi:10.1039/c8cc05721d (2018).

4 Mogilyansky, E. & Rigoutsos, I. The miR-17/92 cluster: a comprehensive update on its genomics, genetics, functions and increasingly important and numerous roles in health and disease. *Cell Death & Differentiation* **20**, 1603-1614, doi:10.1038/cdd.2013.125 (2013).
