# Supplementary material for: Targeting non-coding RNA family members with artificial endonuclease XNAzymes
Source: Commun Biol. 2022 Sep 24;5:1010. doi: 10.1038/s42003-022-03987-5 (PMC9509326; doi:10.1038/s42003-022-03987-5)
Supplement: Supplementary file 2 — Description of Additional Supplementary Data [file 42003_2022_3987_MOESM2_ESM.docx]

**Description of Additional Supplementary Files**

**File name:** Supplementary Data 1

**Description:** Raw gels shown and used to generate graphs in the paper
