# Supplementary material for: Targeting non-coding RNA family members with artificial endonuclease XNAzymes
Source: Commun Biol. 2022 Sep 24;5:1010. doi: 10.1038/s42003-022-03987-5 (PMC9509326; doi:10.1038/s42003-022-03987-5)

Supplementary Data for:

Targeting non-coding RNA family members with artificial endonuclease XNAzymes

Maria J. Donde[1], Adam M. Rochussen[1], Saksham Kapoor[1] and Alexander I. Taylor[1]*

[1]Cambridge Institute of Therapeutic Immunology & Infectious Disease (CITIID), Jeffrey Cheah Biomedical Centre, University of Cambridge, Cambridge, UK.

*corresponding author: ait29@cam.ac.uk

Contents:

1. Raw gel images shown in main figures.
2. Raw gel images used to generate plots shown in main figures.

Raw gel images shown in main figures

Figure 1d

Lane 4: partially hydrolysed miR-17
Lane 5: miR-17 - catalyst
Lane 6: miR-17 + Fz_miR_17

Other lanes: unrelated experiments

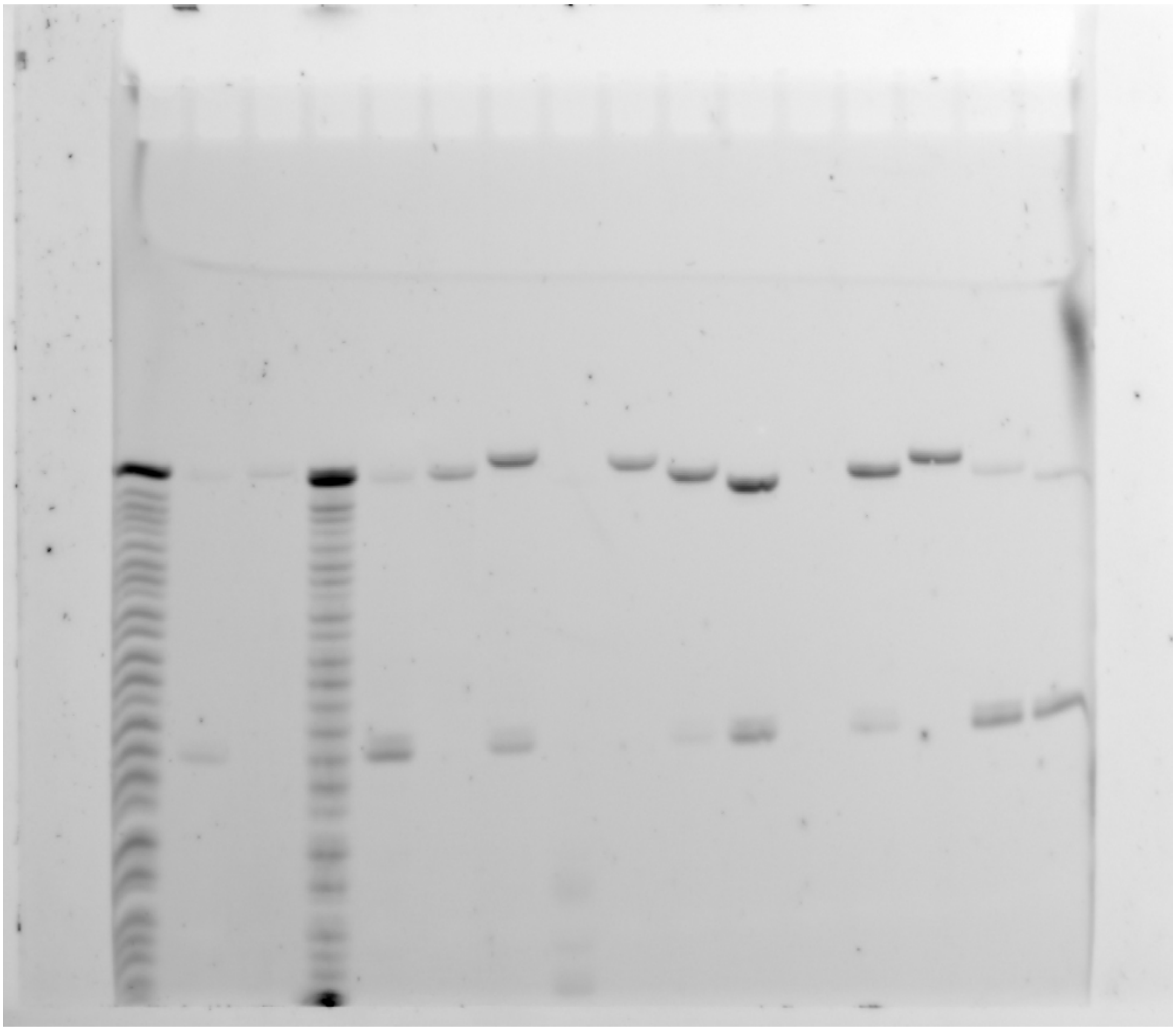

Figure 1f and 1j

Lane 3: miR-17 + Fz_miR_17
Lane 4: miR-20a + Fz_miR_17
Lane 5: miR-20b + Fz_miR_17
Lane 6: miR-21 + Fz_miR_17
Lane 7: miR-93 + Fz_miR_17
Lane 8: miR-106a + Fz_miR_17
Lane 9: miR-106b + Fz_miR_17

Lane 12: miR-17 + Fz_miR_20a
Lane 13: miR-20a + Fz_miR_20a
Lane 14: miR-20b + Fz_miR_20a
Lane 15: miR-21 + Fz_miR_20a
Lane 16: miR-93 + Fz_miR_20a
Lane 17: miR-106a + Fz_miR_20a
Lane 18: miR-106b + Fz_miR_20a

Other lanes: unrelated experiments

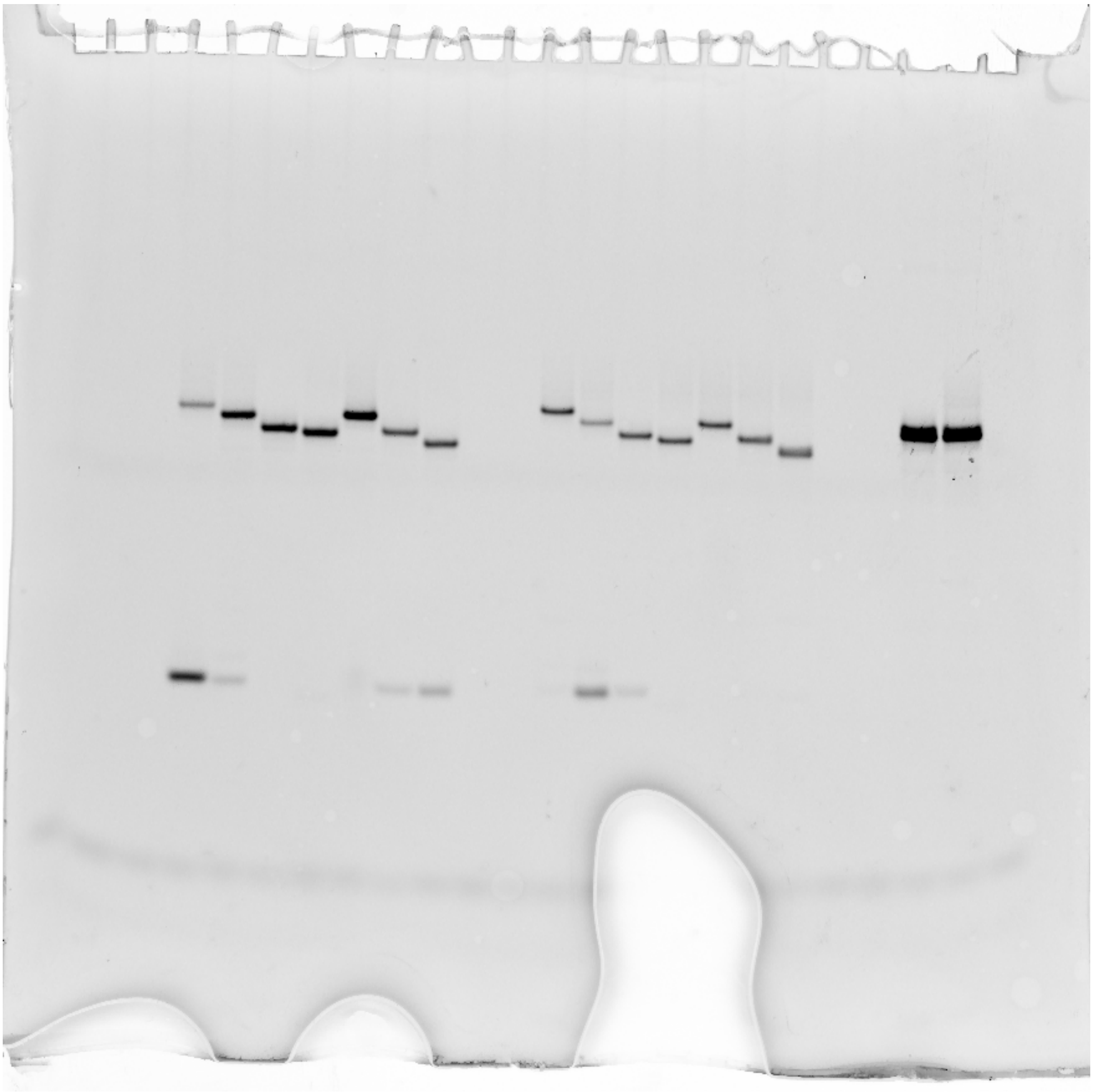

Figure 1h

Lane 13: partially hydrolysed miR-20a
Lane 14: miR-20a - catalyst
Lane 15: miR-20a + Fz_miR_20a

Other lanes: unrelated experiments

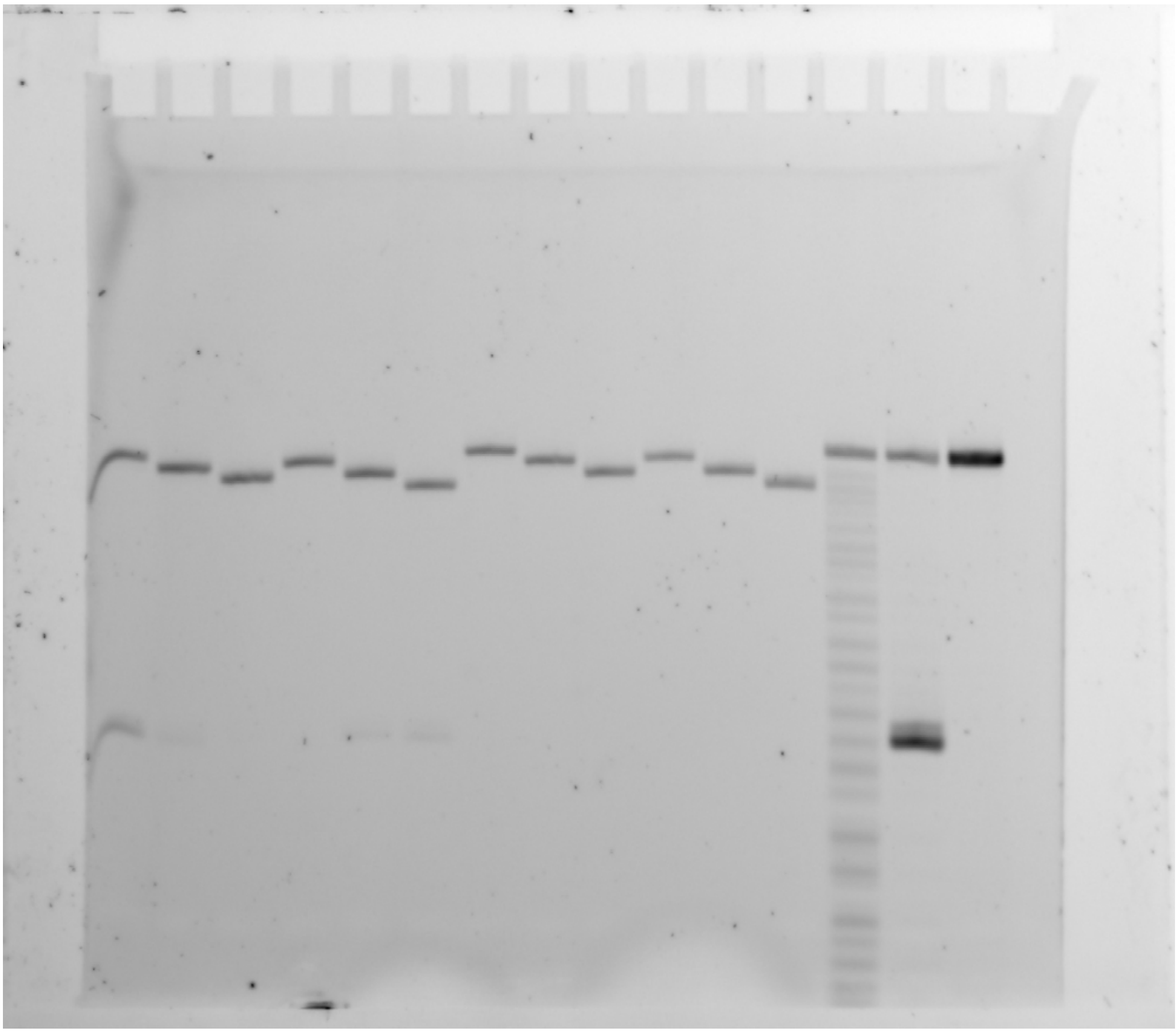

Figure 2b

Lane 4: partially hydrolysed miR-21
Lane 5: miR-21 - catalyst
Lane 6: miR-20 + Fz_miR_21B

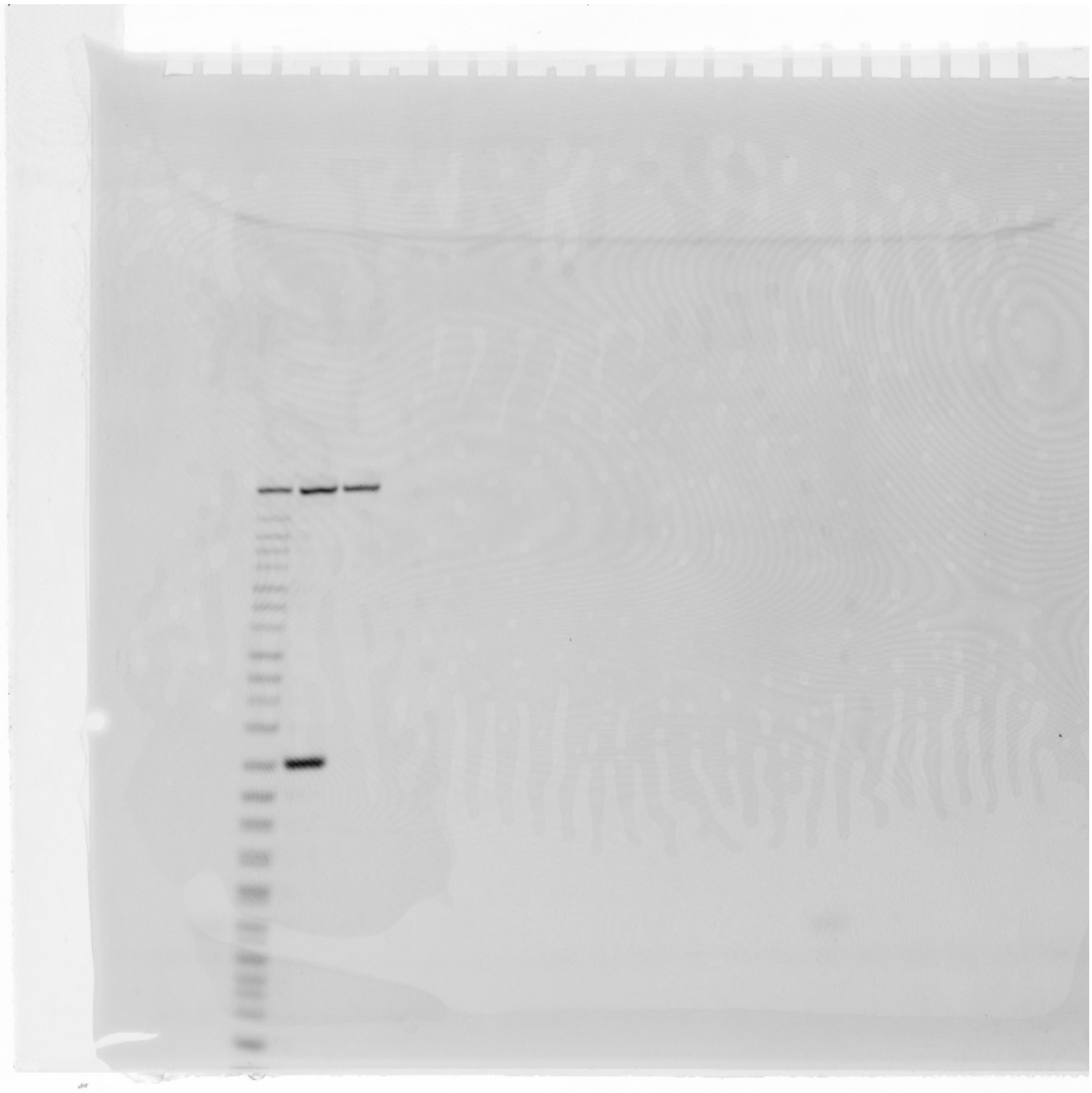

Figure 2d

Lane 17: miR-17 + Fz_miR_21B
Lane 18: miR-20a + Fz_miR_21B
Lane 19: miR-20b + Fz_miR_21B
Lane 20: miR-21 + Fz_miR_21B
Lane 21: miR-93 + Fz_miR_21B
Lane 22: miR-106a + Fz_miR_21B
Lane 23: miR-106b + Fz_miR_21B

Other lanes: unrelated experiments

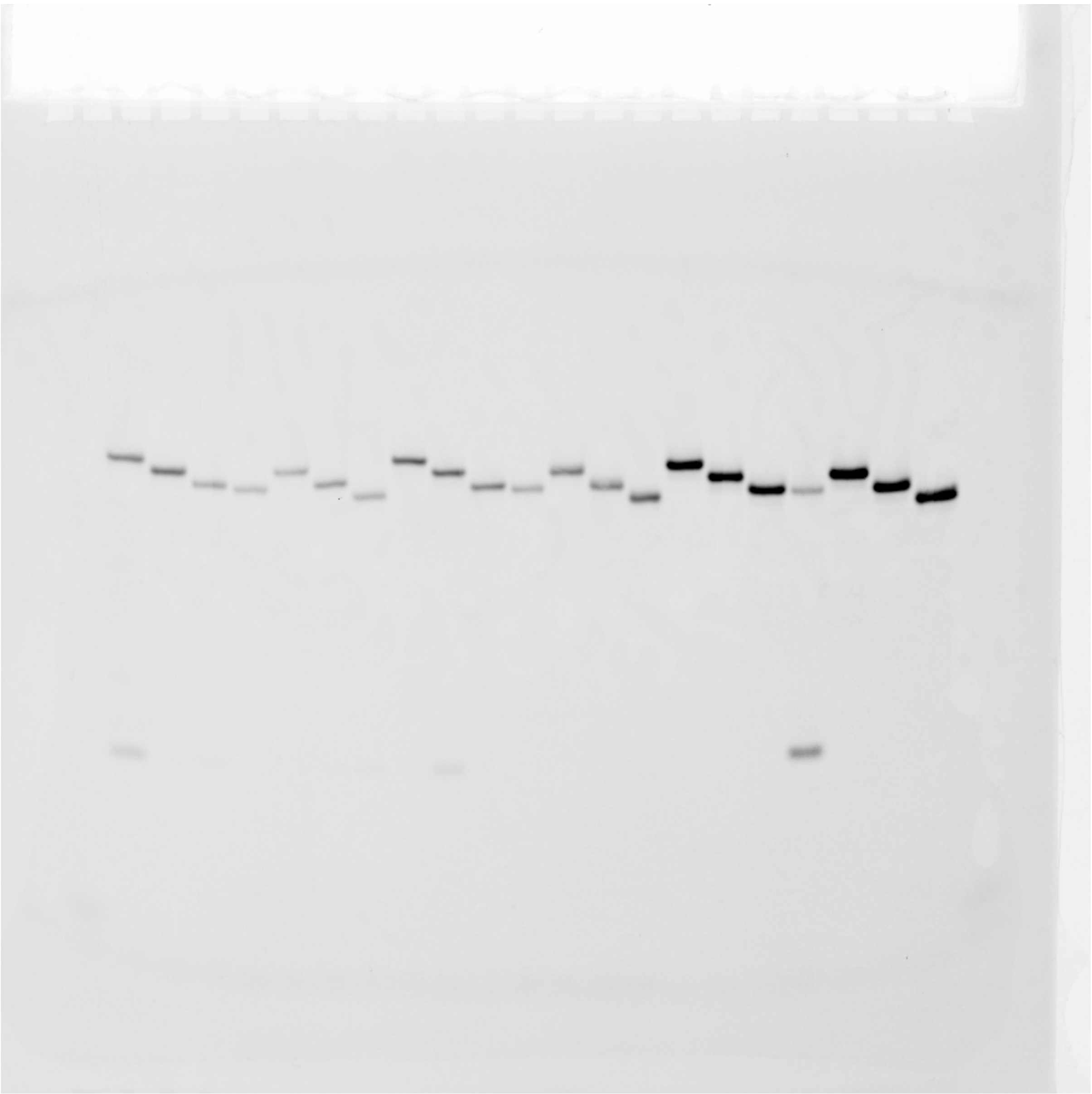

Figure 3d (gel1)

Lane 12: hY1 - catalyst
Lane 13: hY1 + Fz_hY5_1
Lane 14: hY3 - catalyst
Lane 15: hY3 + Fz_hY5_1
Lane 16: hY4 - catalyst
Lane 17: hY4 + Fz_hY5_1
Lane 18: hY5 - catalyst
Lane 19: hY5 + Fz_hY5_1

Other lanes: unrelated experiments

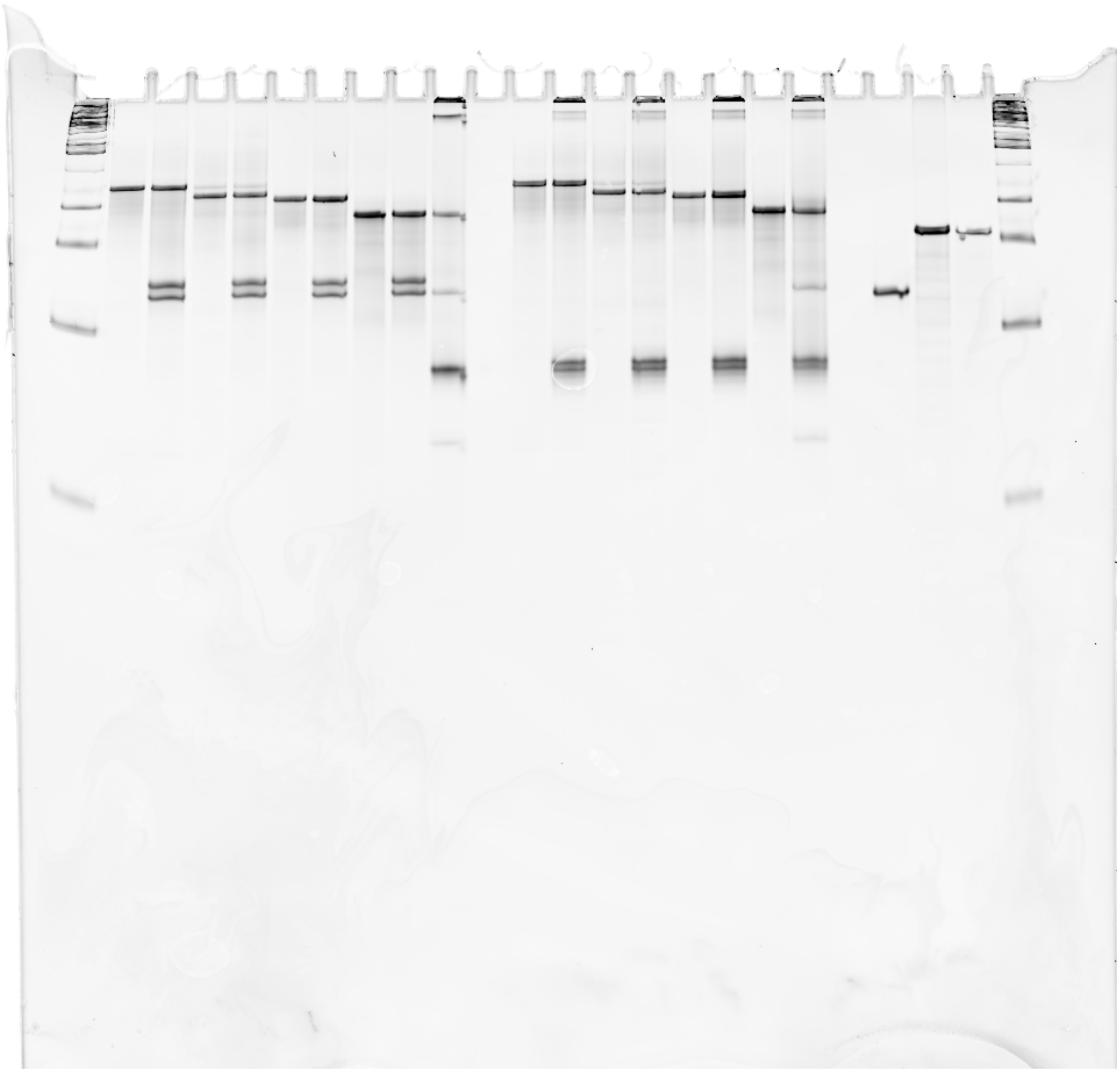

Figure 3d (gel2)

Lane 5: NEB Low molecular weight marker
Lane 7: hY5 + Fz_hY5_1

Other lanes: unrelated experiments

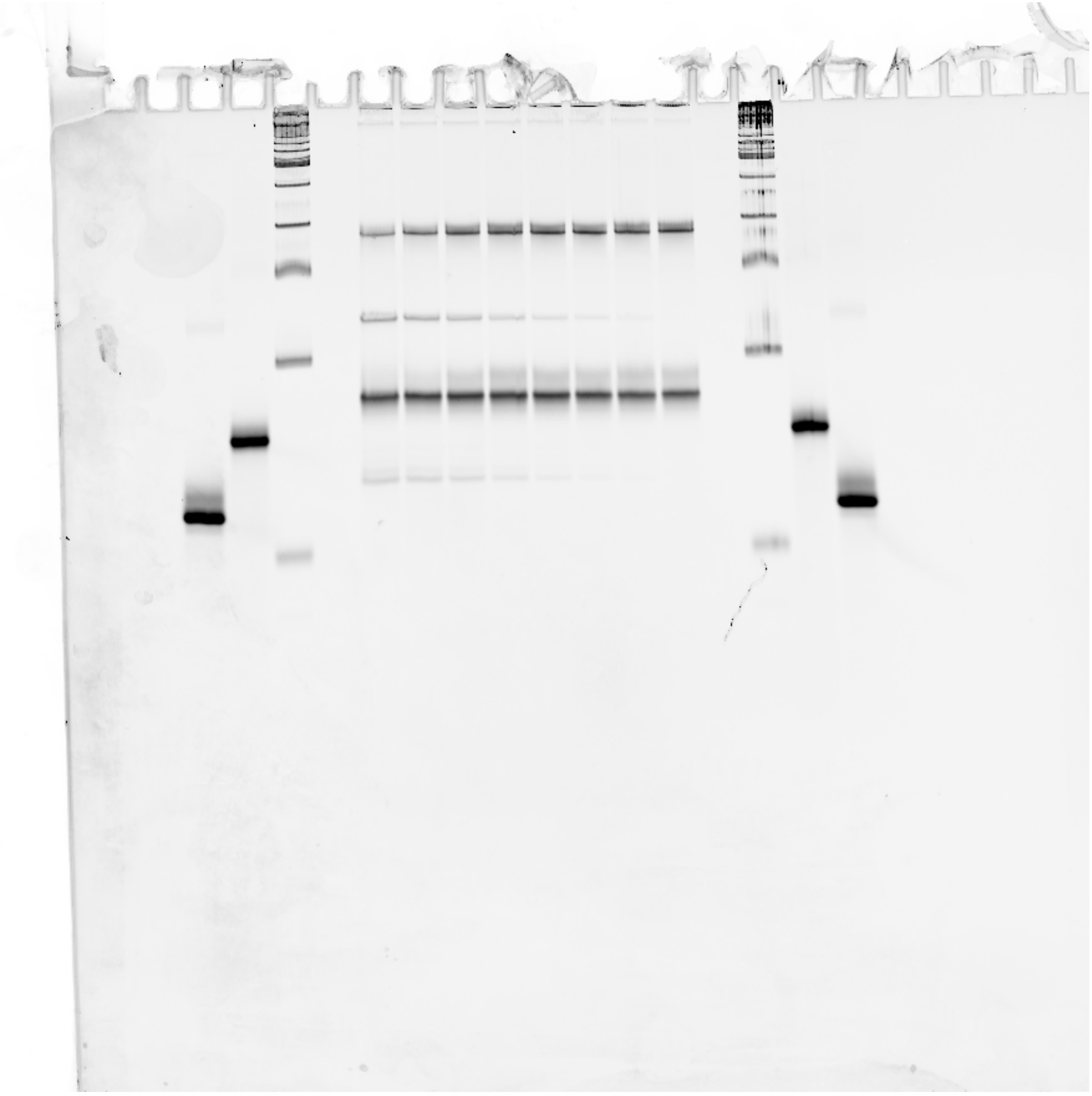

Figure 4b and 4c

Lanes 5, 13, 15 and 18: NEB Low molecular weight marker

Lane 6: TFz_miR_17
Lane 7: TFz_miR_21B
Lane 8: TFz_miR_20a
Lane 9: TFz_miR_17 + TFz_miR_21B
Lane 10: TFz_miR_17 + TFz_miR_20a
Lane 11: TFz_miR_21B + TFz_miR_20a
Lane 12: TFz_miR_17 + TFz_miR_21B + TFz_miR_20a (crude)
Lane 13: NEB Low molecular weight marker

Lane17: TFz_miR_17 + TFz_miR_21B + TFz_miR_20a (filtered)

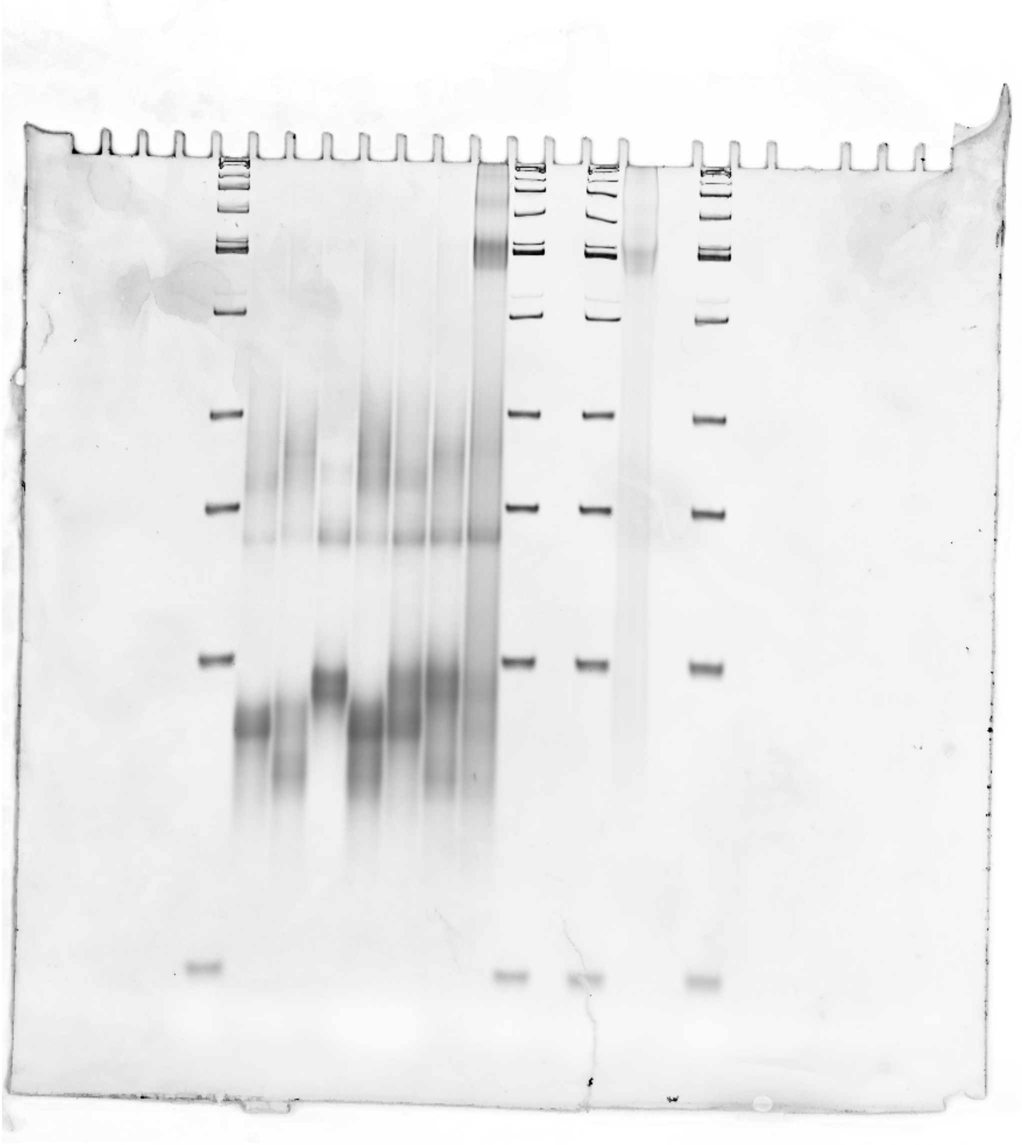

Figure 4e

Lane 2: miR-17 - catalyst
Lane 3: miR-17 + Fz_miR_17
Lane 4: miR-17 + TFz_miR$_3$

Lane 6: miR-20a - catalyst
Lane 7: miR-20a + Fz_miR_20a
Lane 8: miR-20a + TFz_miR$_3$

Lane 10: miR-21 - catalyst
Lane 11: miR-21 + Fz_miR_21B
Lane 12: miR-21 + TFz_miR$_3$

Lane 14: miR-93 - catalyst
Lane 15: miR-93 + TFz_miR$_3$

Other lanes: unrelated experiments

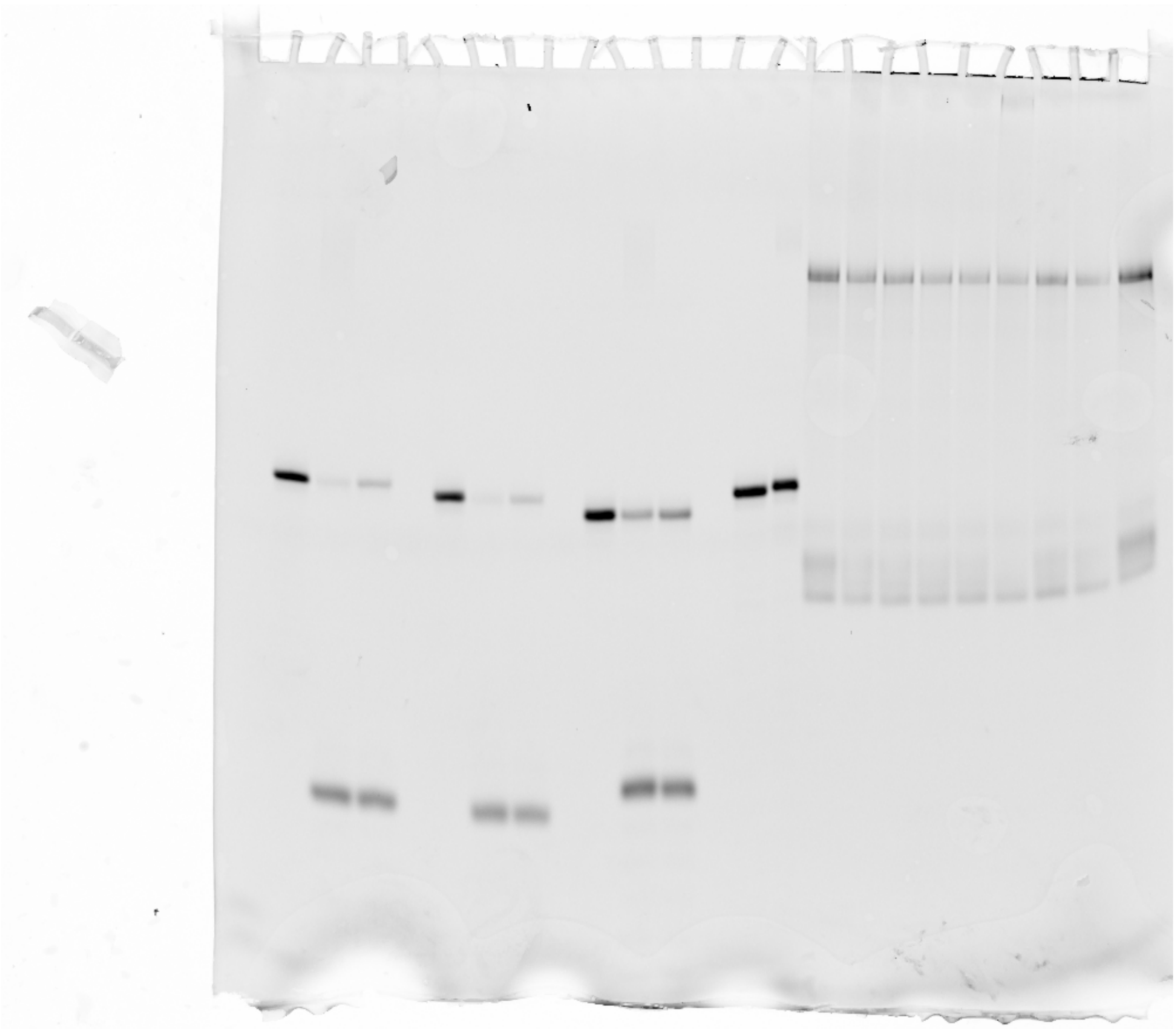

Raw gel images used to generate plots shown in main figures.

Figure 1e

miR-17 + Fz_miR_17 single-turnover timecourse; % cleaved

Lane 1: replicate 1, t = 0 h;	0
Lane 2: replicate 1, t = 4 min;	6
Lane 3: replicate 1, t = 8 min;	8
Lane 4: replicate 1, t = 0.25 h;	11
Lane 5: replicate 1, t = 0.5 h;	22
Lane 6: replicate 1, t = 1 h;	30
Lane 7: replicate 1, t = 2 h;	47
Lane 8: replicate 1, t = 4 h;	ND
Lane 9: replicate 2, t = 0 h;	0
Lane 10: replicate 2, t = 4 min;	5
Lane 11: replicate 2, t = 8 min;	6
Lane 12: replicate 2, t = 0.25 h;	9
Lane 13: replicate 2, t = 0.5 h;	18
Lane 14: replicate 2, t = 1 h;	31
Lane 15: replicate 2, t = 2 h;	47
Lane 16: replicate 2, t = 4 h;	58

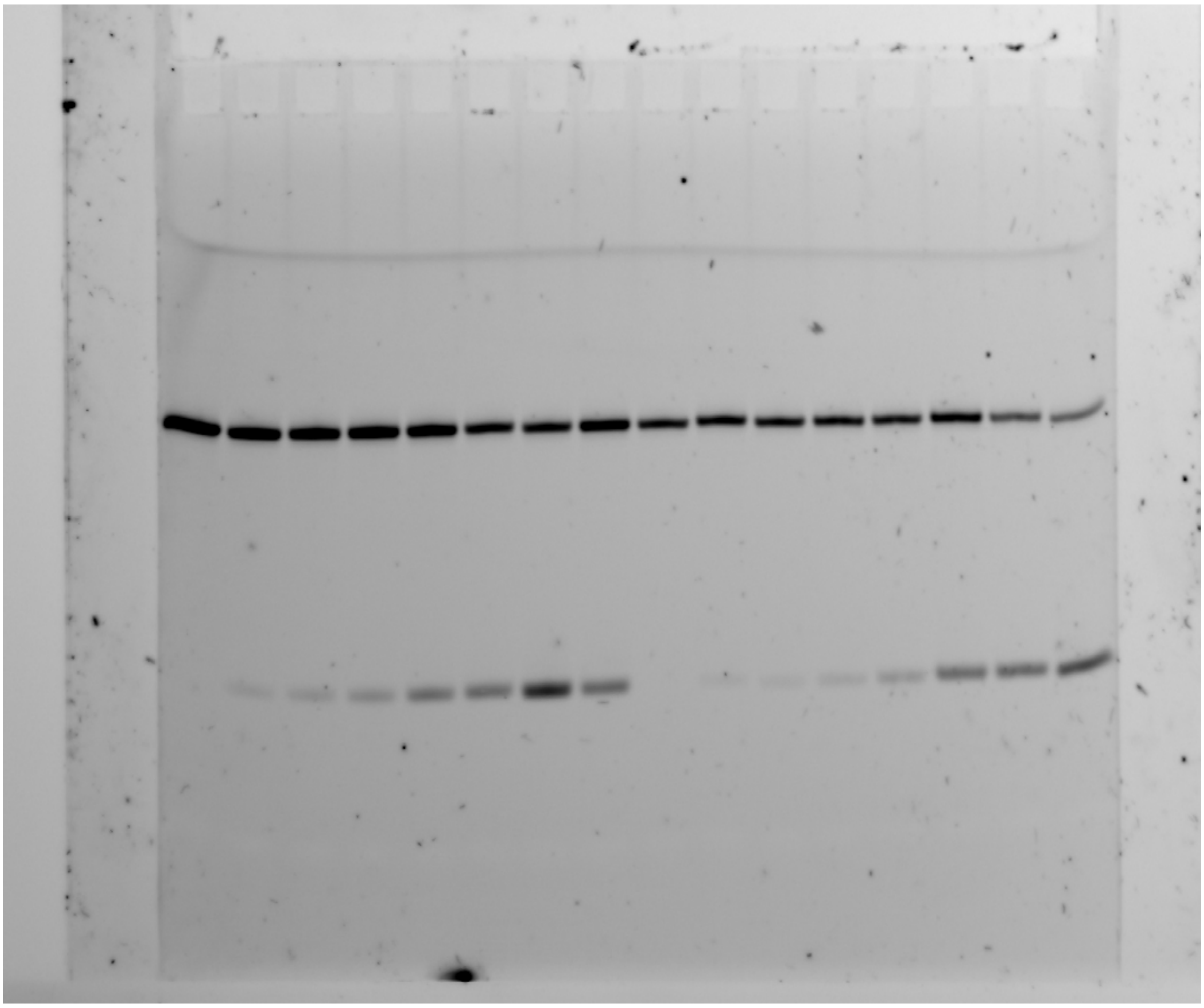

Figure 1e and l

miR-17 + Fz_miR_17 single-turnover timecourse; % cleaved

Lane 1: replicate 3, t = 0 h;	0
Lane 2: replicate 3, t = 8 min;	12
Lane 3: replicate 3, t = 0.25 h;	12
Lane 4: replicate 3, t = 0.5 h;	23
Lane 5: replicate 3, t = 1 h;	35
Lane 6: replicate 3, t = 2 h;	56
Lane 7: replicate 3, t = 4 h;	70
Lane 8: replicate 3, t = 8 h;	79

miR-20a + Fz_miR_20a single-turnover timecourse; % cleaved

Lane 9: replicate 1, t = 0 h;	0
Lane 10: replicate 1, t = 8 min;	8
Lane 11: replicate 1, t = 0.25 h;	13
Lane 12: replicate 1, t = 0.5 h;	18
Lane 13: replicate 1, t = 1 h;	26
Lane 14: replicate 1, t = 2 h;	44
Lane 15: replicate 1, t = 4 h;	61
Lane 16: replicate 1, t = 8 h;	77

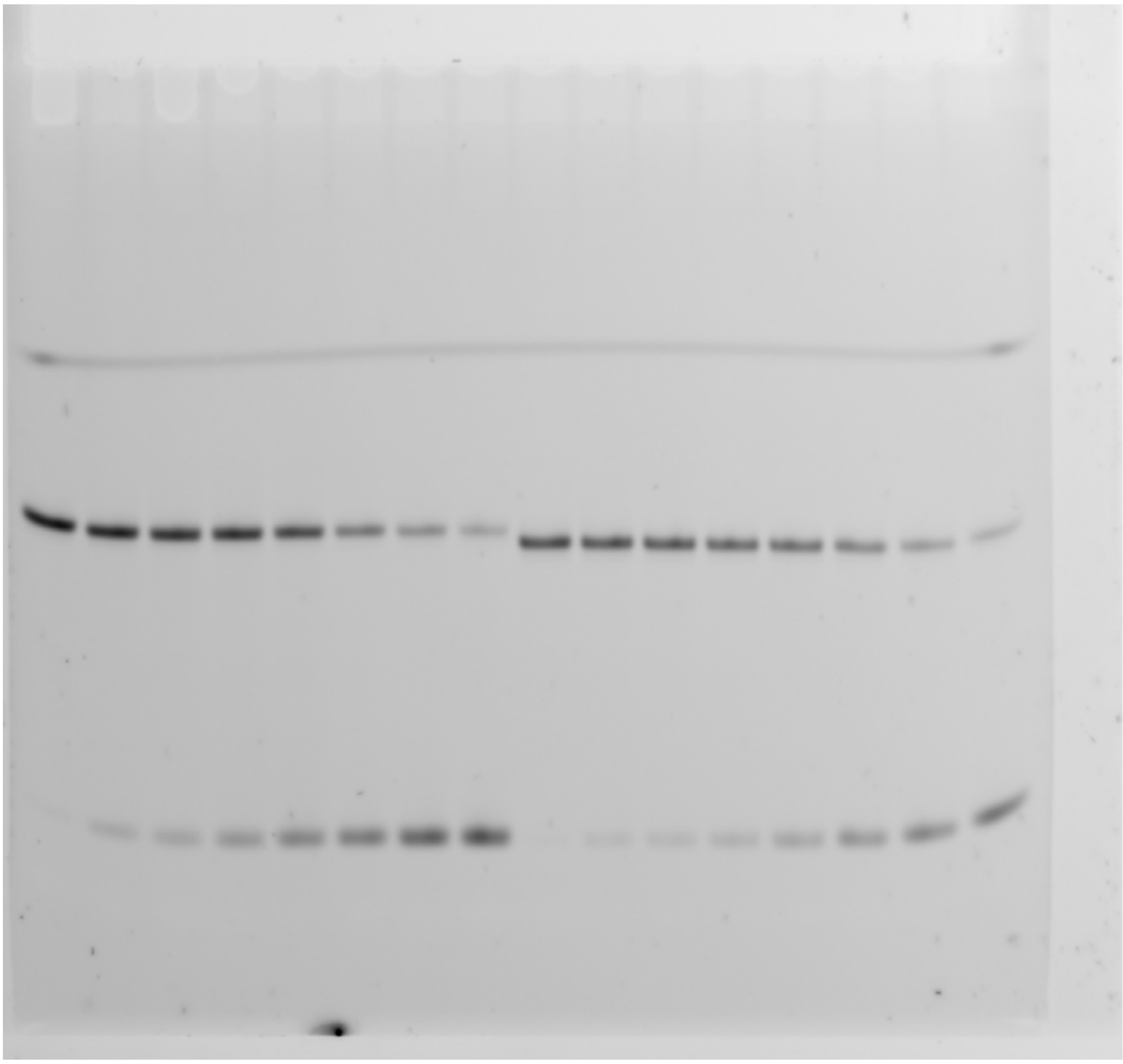

Figure 1e and l

miR-20a + Fz_miR_20a single-turnover timecourse; % cleaved

Lane 1: replicate 2, t = 0 h;	0
Lane 2: replicate 2, t = 8 min;	4
Lane 3: replicate 2, t = 0.25 h;	7
Lane 4: replicate 2, t = 0.5 h;	14
Lane 5: replicate 2, t = 1 h;	19
Lane 6: replicate 2, t = 2 h;	50
Lane 7: replicate 2, t = 4 h;	60
Lane 8: replicate 2, t = 8 h;	78

Lane 10: miR-17 + Fz_miR_17 single-turnover timecourse, replicate 1, t = 8h;	83
Lane 11: miR-17 + Fz_miR_17 single-turnover timecourse, replicate 2, t = 8h;	83
Lane 12: miR-20a + Fz_miR_20a single-turnover timecourse, replicate 3, t = 8h;	80

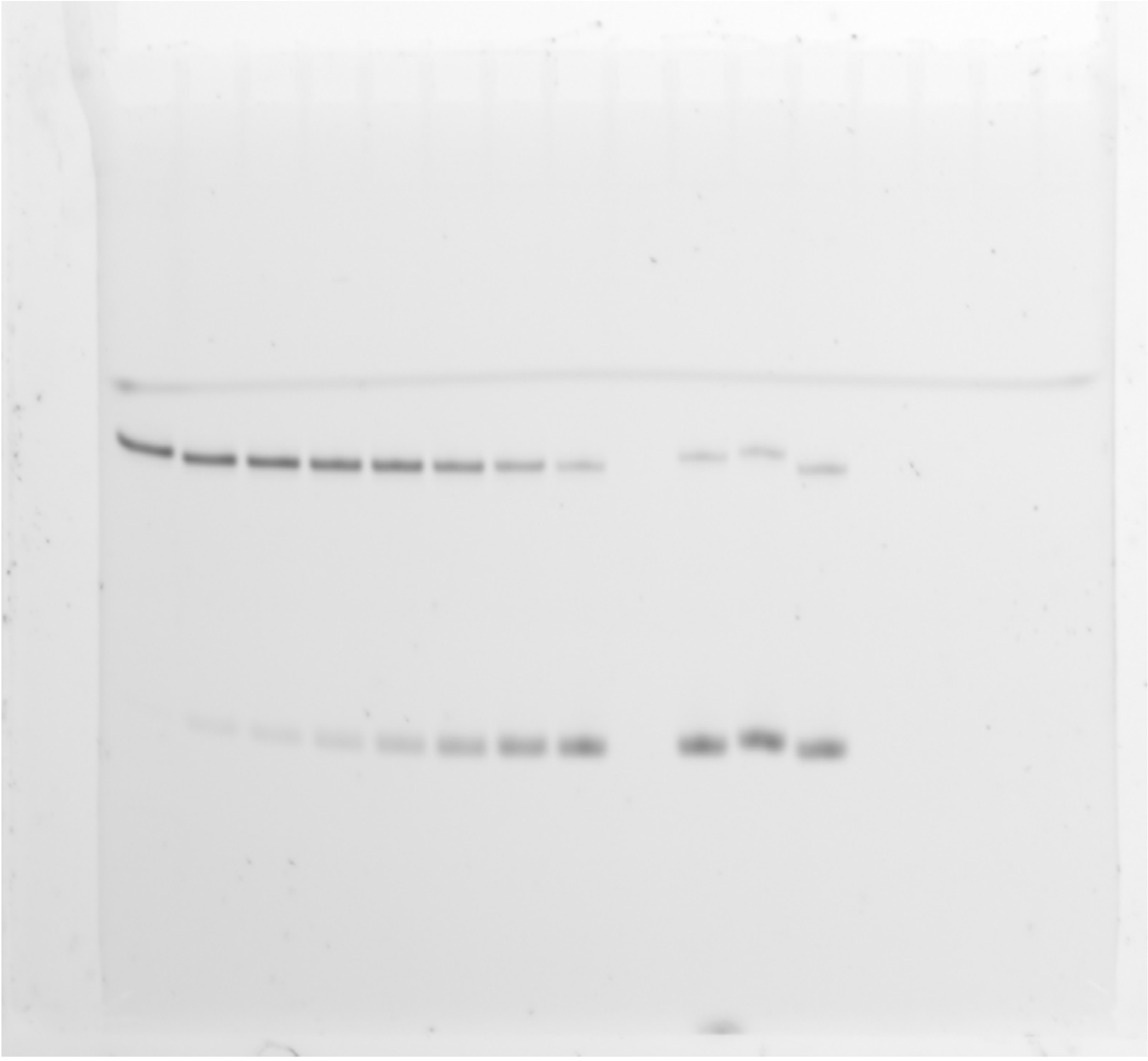

Figure 1i

miR-20a + Fz_miR_20a single-turnover timecourse; % cleaved

Lane 1: replicate 3, t = 0 h;	0
Lane 2: replicate 3, t = 4 min;	ND
Lane 3: replicate 3, t = 8 min;	7
Lane 4: replicate 3, t = 0.25 h;	11
Lane 5: replicate 3, t = 0.5 h;	18
Lane 6: replicate 3, t = 1 h;	29
Lane 7: replicate 3, t = 2 h;	44
Lane 8: replicate 3, t = 4 h;	62

Other lanes: unrelated experiments

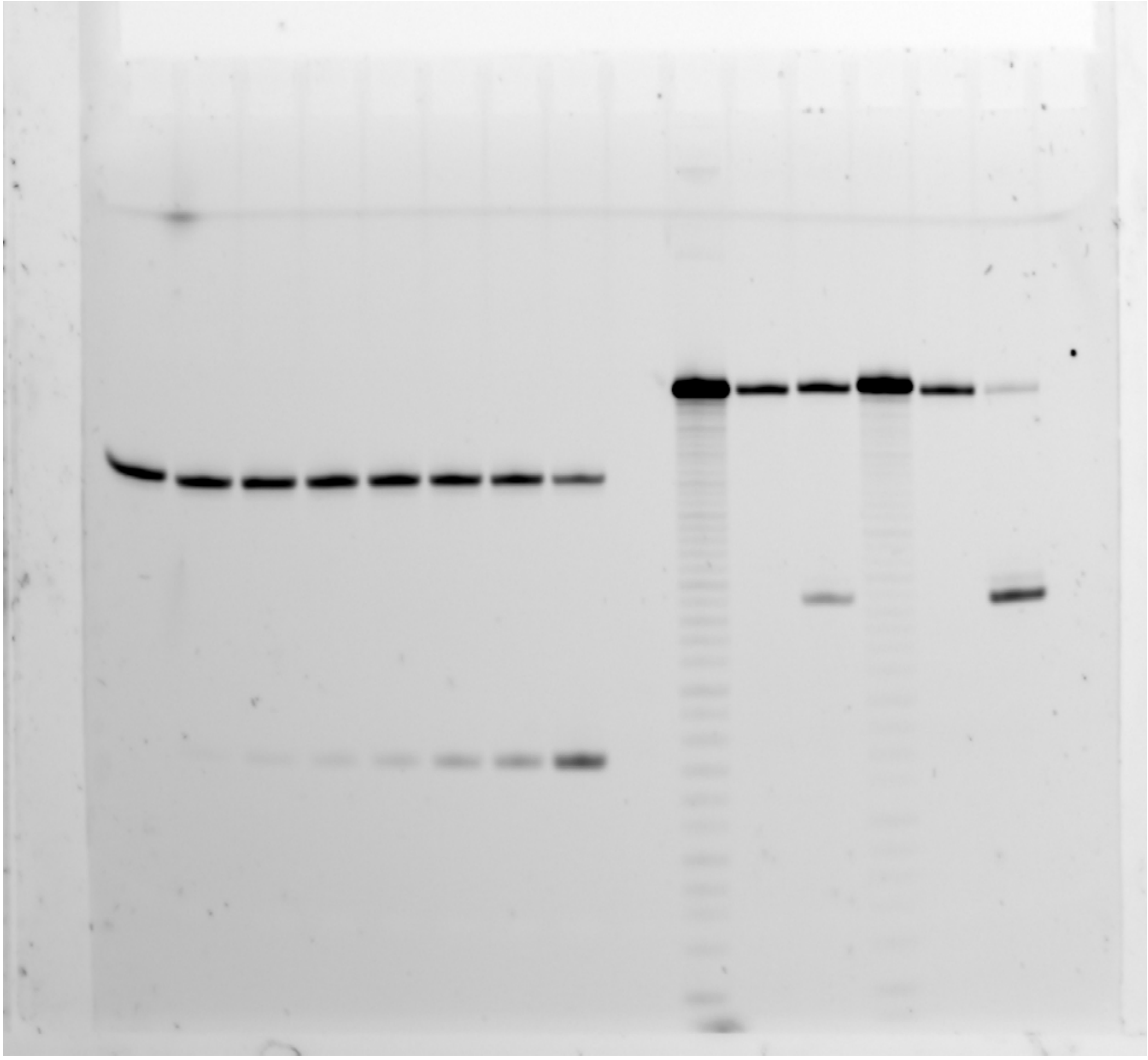

Figure 2c

miR-21 + Fz_miR_21B single-turnover timecourse; % cleaved

Lane 3: replicate 1, t = 0 h;	0
Lane 4: replicate 1, t = 7.5 min;	ND
Lane 5: replicate 1, t = 0.25 h;	8
Lane 6: replicate 1, t = 0.5 h;	10
Lane 7: replicate 1, t = 1 h;	12
Lane 8: replicate 1, t = 2 h;	21
Lane 9: replicate 1, t = 4 h;	30
Lane 10: replicate 1, t = 8 h;	47
Lane 11: replicate 1, t = 16 h;	62

Lane 14: replicate 2, t = 0 h;	0
Lane 15: replicate 2, t = 0.25 h;	5
Lane 16: replicate 2, t = 0.5 h;	10
Lane 17: replicate 2, t = 2 h;	22
Lane 18: replicate 2, t = 4 h;	35
Lane 19: replicate 2, t = 7.5 min;	ND
Lane 20: replicate 2, t = 1 h;	12
Lane 21: replicate 2, t = 8 h;	49
Lane 22: replicate 2, t = 16 h;	62

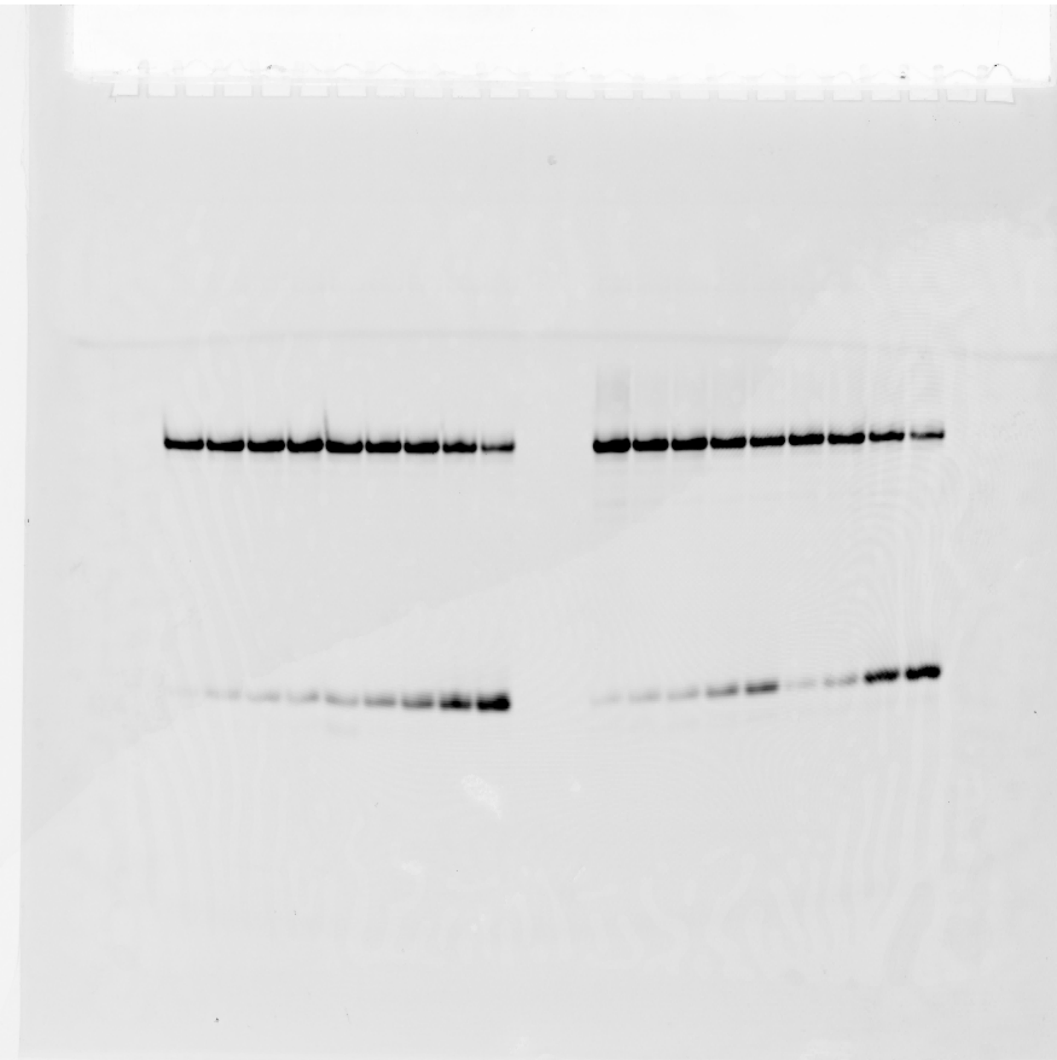

Figure 2c

miR-21 + Fz_miR_21B single-turnover timecourse; % cleavage

Lane 3: replicate 3, t = 7.5 min;	4
Lane 4: replicate 3, t = 0.25 h;	6
Lane 5: replicate 3, t = 0.5 h;	7
Lane 6: replicate 3, t = 1 h;	10
Lane 7: replicate 3, t = 2 h;	15
Lane 8: replicate 3, t = 4 h;	28
Lane 9: replicate 3, t = 8 h;	45
Lane 10: replicate 3, t = 16 h;	67

Other lanes: unrelated experiments

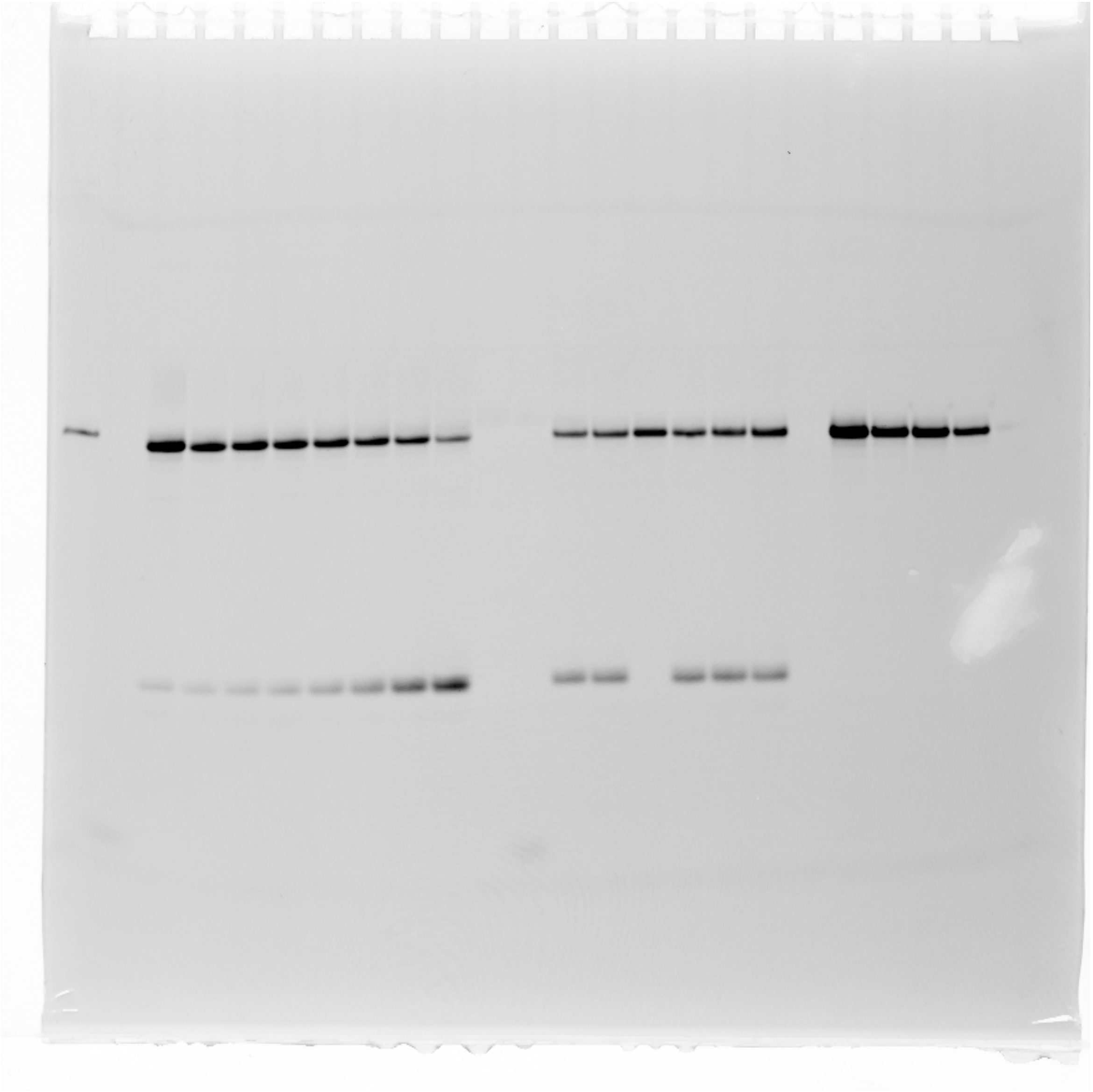

Figure 3b

hY5 + Fz_hY5_4B single-turnover timecourse; % cleavage

Lane 2: NEB Low molecular weight marker
Lane 3: replicate 1, t = 0 h; 0
Lane 4: replicate 1, t = 1.5 h; 4
Lane 5: replicate 1, t = 3 h; 10
Lane 6: replicate 1, t = 6 h; 13
Lane 7: replicate 1, t = 12 h; 22
Lane 8: replicate 1, t = 24 h; 34
Lane 9: replicate 1, t = 48 h; 50
Lane 10: replicate 1, t = 96 h; 61
Lane 12: replicate 2, t = 0 h; 0
Lane 13: replicate 2, t = 1.5 h; 5
Lane 14: replicate 2, t = 3 h; 8
Lane 15: replicate 2, t = 6 h; 14
Lane 16: replicate 2, t = 12 h; 21
Lane 17: replicate 2, t = 24 h; 33
Lane 18: replicate 2, t = 48 h; 45
Lane 19: replicate 2, t = 96 h; 58
Lane 20: NEB Low molecular weight marker

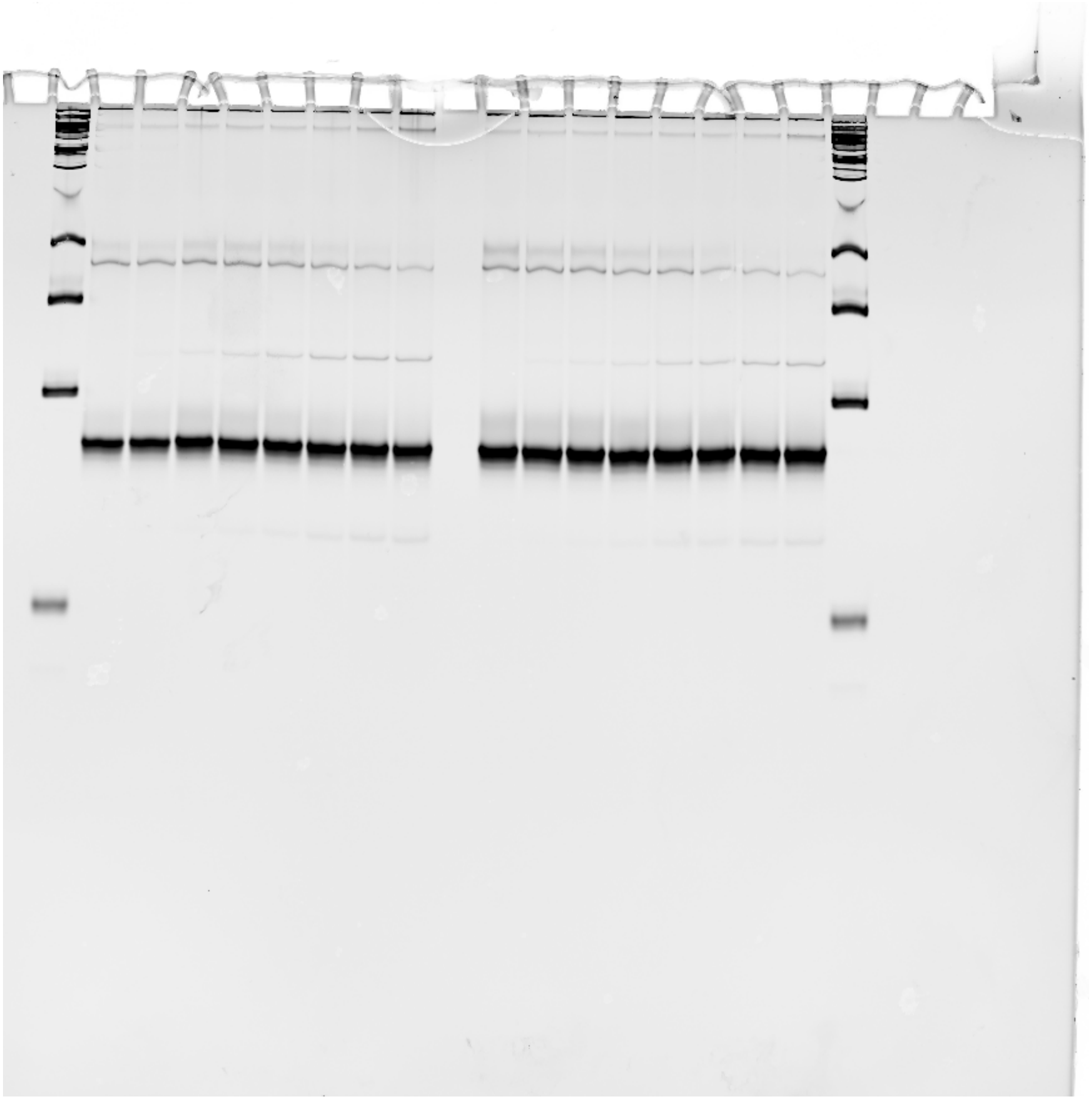

Figure 3b

hY5 + Fz_hY5_4B single-turnover timecourse; % cleavage

Lanes 4 & 9: NEB Low molecular weight marker

Lane 11: replicate 3, t = 0 h;		0
Lane 12: replicate 3, t = 1.5 h;		5
Lane 13: replicate 3, t = 3 h;		8
Lane 14: replicate 3, t = 6 h;		14
Lane 15: replicate 3, t = 12 h;		24
Lane 16: replicate 3, t = 24 h;		38
Lane 17: replicate 3, t = 48 h;		52
Lane 18: replicate 3, t = 96 h;		62

Other lanes: unrelated experiments

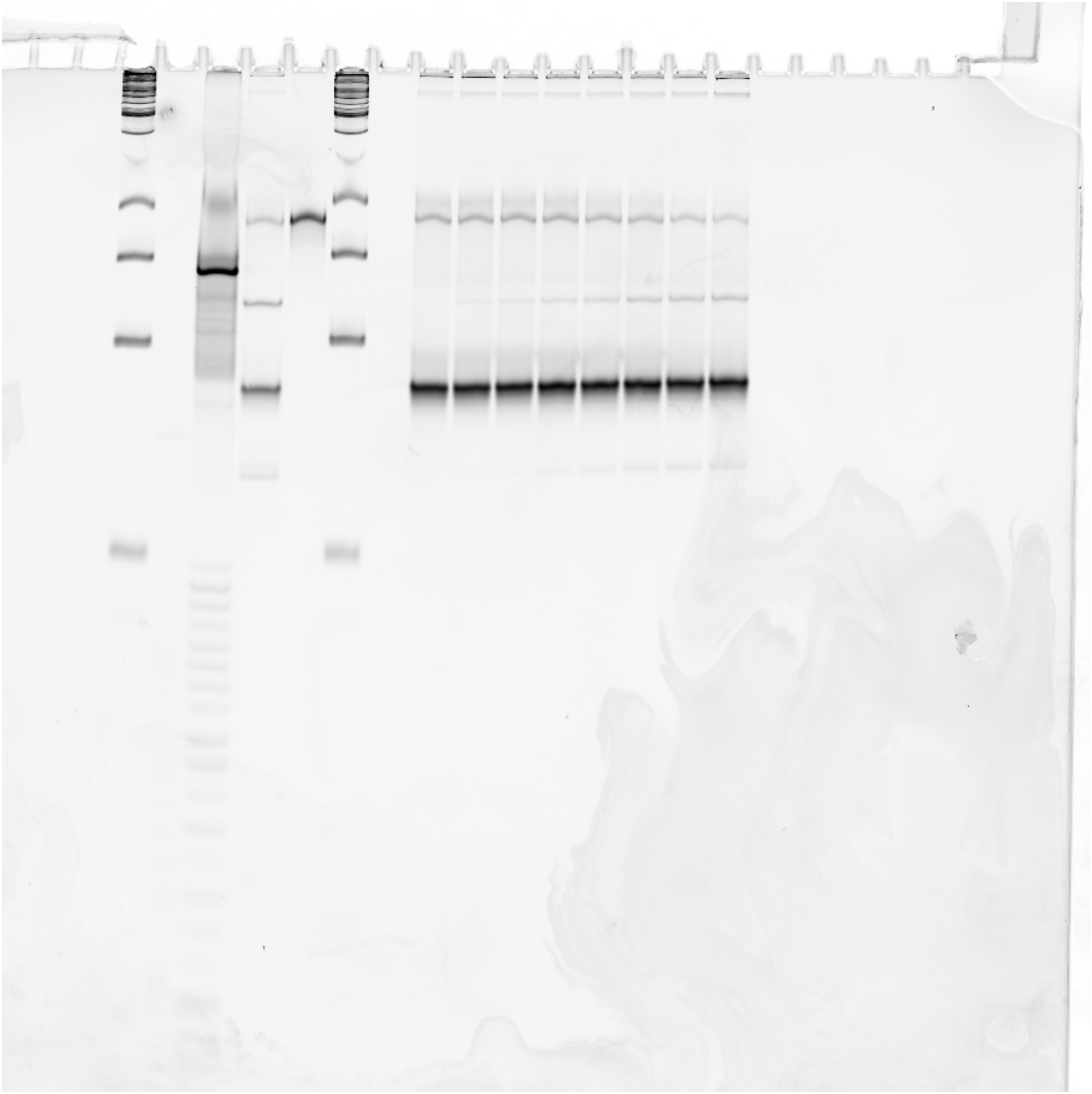

Figure 4d

Serum stability timecourse; % undegraded full-length

Lanes 2, 11 & 20: NEB Low molecular weight marker
Lane 3: Fz_miR_21B, 0 h; 100
Lane 4: Fz_miR_21B, 1 h; 93
Lane 5: Fz_miR_21B, 3 h; 27
Lane 6: Fz_miR_21B, 6 h; 16
Lane 7: Fz_miR_21B, 24 h; 3
Lane 8: Fz_miR_21B, 48 h; 3
Lane 9: Fz_miR_21B, 96 h; 1
Lane 12: TFz_miR$_3$, 0 h; 100
Lane 13: TFz_miR$_3$, 1 h; 92
Lane 14: TFz_miR$_3$, 3 h; 92
Lane 15: TFz_miR$_3$, 6 h; 92
Lane 16: TFz_miR$_3$, 24 h; 55
Lane 17: TFz_miR$_3$, 48 h; 21
Lane 18: TFz_miR$_3$, 96 h; 21

Other lanes: unrelated experiments

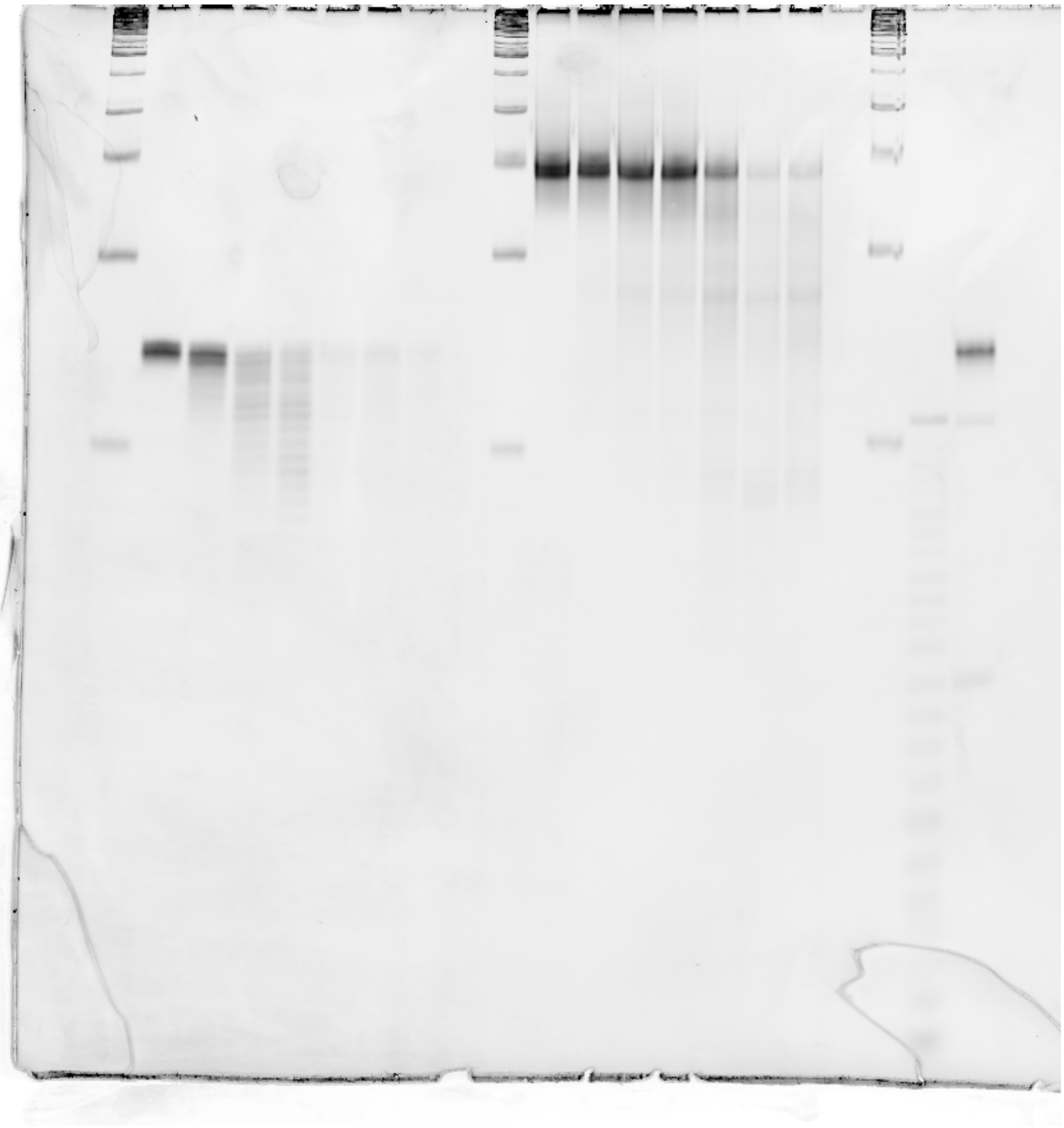

Supplement: Supplementary file 3 — Supplementary Data 1 [file 42003_2022_3987_MOESM3_ESM.pdf]
